# Characterization of the bacteriophage vB_KpnP_Henu1_3 lytic for K1 *Klebsiella pneumoniae* and its therapeutic efficacy in *Galleria mellonella* larvae and mice

**DOI:** 10.1128/spectrum.00931-25

**Published:** 2026-01-06

**Authors:** Yuan Zhang, Lin Shi, Fang Zhou, Jiaqi Li, Mengzhe Liu, Shuai Guo, Xiaoyu Shi, Xinwei Zhang, Dongliang Qiao, Jiangfeng Zhang, Kexiao Wang, Tieshan Teng, Youhua Yuan, Qiming Li, Shanmei Wang

**Affiliations:** 1 Department of Clinical Laboratory, Henan Provincial People's Hospital, People’s Hospital of Zhengzhou University, and People’s Hospital of Henan University 89632, Zhengzhou, China; 2 Henan Province Engineering Technology Research Center of Rapid-Accuracy Medical Diagnostics, Department of Clinical Laboratory, The First Affiliated Hospital of Henan University, Henan University 12411, Kaifeng, China; 3 Department of Microbiology, College of Basic Medical Sciences, Henan University 12411, Kaifeng, China; 4 The Jointed National Laboratory of Antibody Drug Engineering, Henan University 12411, Kaifeng, China; Shandong First Medical University, Jinan, Shandong, China; King Abdulaziz University, Jeddah, Makkah al mukarramah, Saudi Arabia

**Keywords:** bacteriophage, phage therapy, antibiotic resistance, *Klebsiella pneumoniae*, vB_KpnP_Henu1_3

## Abstract

New anti-infective therapies are urgently needed for the treatment of drug-resistant bacterial infections in the context of the rapid spread of drug resistance. Phages, the natural enemies of bacteria, have irreplaceable advantages in the treatment of bacterial infections. Here, we report a novel phage, vB_KpnP_Henu1_3, that specifically lyses capsule-type K1 *Klebsiella pneumoniae*. The phage vB_KpnP_Henu1_3 shows relatively favorable thermal stability (4°C–55°C) and pH tolerance (pH = 4–11). In addition, the optimal multiplicity of infection is 0.01, with a burst size of approximately 253 ± 54 PFU/cell. Genomic analysis reveals that phage vB_KpnP_Henu1_3 contains double-stranded DNA (total length of 49,808 bp) with a G + C content of 50.76%, and the genome comprises 75 open reading frames with no virulence- or antibiotic resistance-related genes. Transmission electron microscopy observations revealed that phage vB_KpnP_Henu1_3 possessed an icosahedral head and siphovirus morphology. Based on the genome sequence, phage vB_KpnP_Henu1_3 could be assigned to a new species in the genus *Webervirus* of the family *Drexlerviridae*. Furthermore, phage vB_KpnP_Henu1_3 rapidly inhibits the growth of *K. pneumoniae* within 4 h, prevents biofilm formation, and disrupts mature biofilms *in vitro*. In infection animal models, phage vB_KpnP_Henu1_3 significantly increases the survival rate of *K. pneumoniae*-infected *Galleria mellonella* larvae and mice while reducing the bacterial loads. These findings demonstrate that phage vB_KpnP_Henu1_3 has promising potential as a safe alternative for controlling and treating multidrug-resistant K1-type *K. pneumoniae* infections.

**IMPORTANCE:**

The widespread use of antibiotics has led to increasing antibiotic resistance, which is a growing global health concern. Therefore, the development of novel antimicrobial therapy that can cure drug-resistant bacteria-induced infections is imperative. Phages are of increasing interest as natural enemies of bacteria with clear advantages in antibacterial.

## INTRODUCTION

*Klebsiella pneumoniae* has emerged as a clinically significant pathogen, accounting for a substantial proportion of both healthcare-associated infections and community-acquired invasive diseases. As a leading etiological agent, it demonstrates particular clinical relevance in hospital settings where it frequently causes pneumonia, bloodstream infections, and urinary tract infections among immunocompromised patients. *K. pneumoniae* has evolved from having typical characteristics to being a highly virulent *K. pneumoniae* (hvKp) and is widely prevalent worldwide (1, 2). With the widespread use of broad-spectrum antimicrobials such as β-lactams and aminoglycosides, *K. pneumoniae* is prone to produce extended-spectrum β-lactamases (ESBLs) and cephalosporinases (AmpC), as well as aminoglycoside-modifying enzymes, and exhibits severe multidrug resistance to commonly used drugs, including third-generation cephalosporins and aminoglycosides (3–6). The prevalence of multidrug resistance genes has been accompanied by an increasing number of multidrug-resistant, highly virulent *K. pneumoniae* strains (7). The most common serotypes of hvKp include K1, K2, K20, K54, and K57, with K1 and K2 being the most virulent and accounting for 70% of hvKp isolates (8). The prevalence of the mobile colistin resistance 1 plasmid has also allowed *K. pneumoniae* to break through the last resort of antibiotics against negative bacterial infections, colistin (9). Cases of hospital-acquired infections caused by *K. pneumoniae* have been increasing annually. Additionally, the increasing number of multidrug-resistant strains frequently leads to the failure of clinical antimicrobial therapy and the prolongation of disease. There is an imperative clinical need for innovative therapeutic approaches for the management of *K. pneumoniae* infections (10).

Phages, which serve as the natural predators of bacteria, have irreplaceable advantages over antimicrobial agents and are employed in the treatment of bacterial infections (11). Currently, with the widespread prevalence of drug-resistant bacterial infections, the clinical use of phage therapy has witnessed an increase (12). The pathways by which antibiotics and phages cause bacterial death are completely different, so bacteria that are resistant to antibiotics do not affect bacterial susceptibility to phages (13). There have been many reports of effective phage therapy for *K. pneumoniae* infection. For example, Gina et al. administered intravenous phage therapy to a 62-year-old patient with persistent infection of the right prosthetic knee caused by the *K. pneumoniae* complex, and this therapy resulted in the resolution of local symptoms and signs of infection (14). Mario et al. used a lytic bacteriophage preparation to treat a patient with *K. pneumoniae* infection that produced multidrug-resistant carbapenemase (KPC-3) and resulted in the eradication of the microorganism without adverse effects (15). Bacteria have developed an astonishing array of strategies to cope with phage threats at each step of the infection process. When bacteria are infected, they can defend against phages by preventing phage adsorption, preventing phage DNA entry, cutting phage nucleic acids, and activating abortive infection systems (16). Of course, bacteria that have been in contact with one phage for a long time promise to foster adaptive evolution and thus be resistant to the phage. However, this adaptive evolution will come at a cost in terms of renewed sensitivity to antibiotics. Therefore, the combined use of antibiotics and phages is also an effective way to address drug-resistant bacterial infections and combat the emergence of bacterial drug resistance. Saskia et al*.* reported that a 58-year-old kidney transplant patient infected with ESBL-positive *K. pneumoniae* had no response to repetitive treatment with meropenem but was successful with a combination of meropenem and phages (17).

Host specificity and the generation of phage resistance limit the clinical application of phage therapy. The widespread presence of phages in the environment provides many raw materials for phage cocktails. Therefore, the establishment of phage libraries by the isolation and identification of different species of phages and the study of their characteristics are important for the future clinical application of phages. Phages infecting *K. pneumoniae* have been isolated from a variety of environments, including sewage, sludge, and infected samples from animals and humans. However, the number of *K. pneumoniae* phages that have been isolated and characterized is insufficient for future clinical applications. Therefore, it is imperative to isolate a large number of phages as stockpiles for the treatment of future clinically resistant bacterial infections. Hospitals, as aggregation and transmission transit sites for drug-resistant bacteria, may harbor large numbers of phages capable of infecting drug-resistant *K. pneumoniae*.

In this study, we isolated a *K. pneumoniae* phage, named vB_KpnP_Henu1_3, from sewage samples from Kaifeng pulmonary hospital. Biological characteristics, genomic features, and antimicrobial efficacy *in vitro* and *in vivo* were evaluated to determine the application value of vB_KpnP_Henu1_3. Our results indicated that phage vB_KpnP_Henu1_3 has excellent antimicrobial capacity both *in vitro* and *in vivo* and has great potential for clinical application.

## MATERIALS AND METHODS

### Phage isolation and purification

The bacteriophage was isolated following a previously described method with modifications (18). Briefly, untreated sewage collected from Kaifeng Pulmonary Hospital was subjected to initial centrifugation and filtration through a 0.22 μm membrane. The resulting supernatant was combined with *K. pneumoniae* strain Kp1049 (isolated in December 2023, accession number CCTCC PB 2025034) and 0.7% soft agar, which was then overlaid onto LB solid medium. After 24 h of incubation, a single, well-isolated phage plaque was aseptically picked and eluted in PBS buffer. The eluate was subsequently spotted onto a fresh lawn of *K. pneumoniae* Kp1049 for successive rounds of plaque purification. This isolation process was repeated five times to ensure homogeneity and stability of the phage population. Through this stringent purification protocol, a novel lytic bacteriophage specific to *K. pneumoniae* was successfully isolated and designated as *Klebsiella* phage vB_KpnP_Henu1_3.

### Transmission electron microscopy analysis

The morphology of purified phage vB_KpnP_Henu1_3 was examined by negative-stain transmission electron microscopy (TEM). Briefly, 20 μL of high-titer phage lysate was adsorbed onto a carbon-coated copper grid (300 mesh) for 10 min at room temperature. Excess liquid was carefully blotted away, and the grid was negatively stained with 2% (wt/vol) uranyl acetate for 90 s. After air-drying, samples were imaged using a Hitachi TEM system operating at an accelerating voltage of 80 kV.

### Host range of phage vB_KpnP_Henu1_3

Twenty-seven clinical isolates of *K. pneumoniae* and other gram-negative bacteria were collected from various hospital ward environments. The detailed information of all clinical isolates of *K. pneumoniae* is listed in Table S1. All isolates were cultured to the mid-exponential phase prior to analysis. For phage susceptibility testing, bacterial cultures were mixed with 0.7% molten soft agar and overlaid onto LB agar plates to create double-layer agar assays. Ten-fold serial dilutions of phage suspension were spotted onto the prepared bacterial lawns, followed by incubation at 37°C for 12 h. Plaque formation was then assessed to determine phage susceptibility. Efficiency of plating (EOP) is calculated in percent as the PFU/mL of the phages on the test strain divided by the PFU/mL obtained on strain Kp1049 multiplied by 100.

### Determination of optimal multiplicity of infection

Bacterial cells were cultured to the logarithmic phase (OD_600_≈0.5), harvested, and resuspended in fresh LB medium at an initial concentration of 1 × 10^8^ CFU/mL. Phage vB_KpnP_Henu1_3 was then added at varying multiplicity of infections (MOIs; 100, 10, 1, 0.1, 0.01, 0.001, and 0.0001), followed by a 10-min adsorption period at 37℃ with gentle agitation. Unadsorbed phages were removed by centrifugation (9,000 × *g*, 5 min), and the infected bacterial pellets were resuspended in 5 mL of fresh LB medium. The cultures were further incubated with shaking (200 rpm) at 37°C for 2 h to allow phage replication. After incubation, the samples were centrifuged (12,000 × *g*, 10 min), and the supernatants were filtered (0.22 μm pore size) to remove residual bacterial cells. Finally, phage titers were determined via the standard double-layer plaque assay.

### Phage adsorption assay

The adsorption kinetics were analyzed using a modified version of established protocols (18). Briefly, *K. pneumoniae* Kp1049 was grown to the mid-logarithmic phase (OD₆₀₀ ≈ 0.5) prior to infection. Phage particles were introduced at an MOI of 0.01 and incubated with shaking at 37°C. Aliquots (200 μL) were collected at defined intervals (0, 2, 4, 6, 8, 10, and so on), immediately centrifuged (12,000 × *g*, 2 min), and the supernatant was serially diluted in PBS. Residual unadsorbed phage titers were determined in triplicate using the double-layer agar method. The adsorption rate was calculated as follows: Unadsorption rate (%) = [(free phage titer)/(initial phage titer)] × 100.

### One-step growth curve analysis

The one-step growth curve was determined to measure the incubation period and the burst size of the phage as previously reported with some modifications (19, 20). Briefly, exponential-phase *K. pneumoniae* Kp1049 (1 × 10^8^ CFU/mL) was infected at an MOI of 0.1 and incubated at 37°C for 10 min to allow phage adsorption. Following a brief centrifugation (4,000 × *g*, 5 min, room temperature) to remove unadsorbed phages, the pellet was resuspended in pre-warmed LB broth at a 1:1,000 dilution. The infected culture was then incubated at 37°C with shaking (180 rpm). Aliquots were collected at 10-min intervals over a 120-min period, immediately centrifuged (12,000 × *g*, 1 min) to separate phage particles from bacterial debris. Phage titers at each time point were subsequently quantified using the standard double-layer agar plaque assay. The experiment was repeated on at least three separate occasions. Phage burst size was calculated as described previously (20).

### Temperature and pH stability determination

To measure the stability of the phage, the titer of phage vB_KpnP_Henu1_3 at different pH values and temperatures was determined. For temperature stability, 100 μL of purified high-titer phages was incubated in a water bath at 4°C, 25°C, 37°C, 45°C, or 55°C for 12 h. Then, the phage titer was determined via the double-layer plate method. For pH stability, equal amounts of phage vB_KpnP_Henu1_3 were incubated at pH values of 2, 3, 4, 5, 6, 7, 8, 9, 10, 11, and 12. The pH stability of phages was assessed by incubating them in PBS solutions adjusted to specific pH levels using hydrochloric acid (HCl). Briefly, 1% (vol/vol) of phage suspension was added to each pH-adjusted PBS solution and incubated at 4°C for 12 h. The remaining phage titers were then determined using the double-layer agar method. All the experiments were repeated three times.

### Phage DNA isolation and sequencing

The genomic DNA of phage vB_KpnP_Henu1_3 was extracted using a standard protocol as previously described (21, 22). Briefly, the purified phage vB_KpnP_Henu1_3 was incubated with DNase I (5 μg/mL) and RNaseA (1 μg/mL) to remove the host DNA. Subsequently, EDTA (pH = 8.0, 20 mM), proteinase K (50 μg/mL), and SDS (0.5% vol/vol) were added to digest the nucleocapsid of the phage and release the phage DNA. The DNA was then purified using Tris-phenol and chloroform, followed by ethanol precipitation. Finally, the pellet was dissolved in distilled water used for DNA sequencing. A paired-end 150 bp DNA library was subsequently constructed via the TruSeqTM DNA Sample Prep Kit according to the manufacturer’s instructions, and the phage vB_KpnP_Henu1_3 genome was sequenced on the Illumina NovaSeq platform. The raw sequencing data were filtered for low-quality reads (≤50 bp) and adapters via Trimmomatic 0.36 with default parameters (23). The quality threshold applied for retaining reads (including those longer than 50 bp) requires an average Q value of ≥20 across 5-bp sliding windows. This criterion ensures that the filtered reads not only satisfy the length requirement but also correspond to a sequencing error rate of ≤1%. Finally, the clean reads were assembled to form a 49,808 bp circular contig via A5-MiSeq and SPAdes software (24, 25).

### Bioinformatics analysis

Open reading frames (ORFs) were predicted via Softberry (http://www.softberry.com/). The ORFs were annotated via the protein basic local alignment search tool (BLASTp) of the National Center for Biotechnology Information (NCBI) server with standard databases. The putative transfer RNA genes in the phage vB_KpnP_Henu1_3 genome were determined by tRNAscan-SE (26). The possible virulence and pathogen genes carried by the phage genome were predicted with VirulenceFinder. Antimicrobial resistance genes and lifestyle traits were predicted using PhageScope (27). The classification of phage vB_KpnP_Henu1_3 was performed using the TaxMyPhage tool in accordance with ICTV guidelines (28). Graphical maps of the annotated phage vB_KpnP_Henu1_3 genome were prepared using Proksee (29).

### Comparative genome analysis

For multiple comparisons, phage genomic sequences exhibiting similarity to vB_KpnP_Henu1_3 were identified and subjected to sequence similarity analysis using NCBI BLASTn (https://blast.ncbi.nlm.nih.gov/). Comparative genomes were mapped by the Easyfig 2.2.3 tool program.

### Bacteriolytic activity *in vitro*

One milliliter of *K. pneumoniae* Kp1049 in the exponential growth phase was transferred into 50 mL of LB medium. Phage vB_KpnP_Henu1_3 was added with MOIs of 1000, 100, 10, 1, 0.1, and 0.01 to infect *K. pneumoniae* Kp1049. The uninfected culture served as a positive control. The samples were collected every 0.5 h, and the OD_600_ was measured via an ultraviolet spectrophotometer. To evaluate the inhibitory effects of phage vB_KpnP_Henu1_3 on biofilm formation, overnight cultures of *K. pneumoniae* Kp1049 were adjusted to 10^7^ CFU/mL in fresh LB medium. Bacterial suspensions were mixed with phage vB_KpnP_Henu1_3 at MOIs of 100, 10, 1, 0.1, and 0.01. Following a 10-min adsorption at 37°C, 200 µL aliquots were transferred to a sterile 96-well microplate (*n* = 3). 200 µL bacterial suspension without phage as the positive control. Plates were incubated statically for 12 h at 37°C. Biofilm formation was quantified using crystal violet staining (G1059, Solarbio) as previously described (30). To assess phage-mediated disruption of established biofilms, mature biofilms were formed by incubating 200 µL aliquots of *K. pneumoniae* Kp1049 (10^7^ CFU/mL) in 96-well plates for 24 h at 37°C. Supernatants were carefully removed, then wells were gently washed twice with PBS to remove non-adherent cells. Phage suspensions (200 μL) at the same MOIs used in inhibition assays were added to respective wells (*n* = 3). Biofilms without phage treatment served as the positive control. Plates were re-incubated for 12 h at 37°C, and biofilm biomass was quantified using the same crystal violet staining protocol. All experiments were performed with three independent biological replicates.

### Evaluation of antibacterial ability in *G. mellonella* larvae

The *G. mellonella* larvae were chosen as a model to evaluate the antibacterial effect of vB_KpnP_Henu1_3. First, active *G. mellonella* larvae of approximately 0.3–0.5 g were selected and injected with 5 μL of *K. pneumoniae* Kp1049 at different concentrations into the last remaining leg to determine the appropriate infection dose. Ten larvae were delivered to each group, and three parallel experiments were conducted. *K. pneumoniae* Kp1049 was washed with saline and then diluted to 1 × 10^9^ CFU/mL. After 1 h of bacterial infection, 5 μL of the phage vB_KpnP_Henu1_3 suspension was injected with the MOIs of 100, 10, 1, 0.1, and 0.01, and then incubated at 37°C in the dark. The *G. mellonella* larvae infected with *K. pneumoniae* Kp1049 and treated with PBS served as the negative control group. The survival rate of each group was recorded every 24 h for 7 days. For bacterial loads, 5 μL of a 1 × 10^8^ CFU/mL bacterial suspension was injected to construct the *G. mellonella* larval infection model (*n* = 6), and 5 μL of the phage vB_KpnP_Henu1_3 suspension was injected with MOIs of 100, 10, and 1 after 1 h of bacterial infection. The bacteria were incubated at 37°C in the dark for 24 h, and the bacterial loads were calculated as CFUs.

### Evaluation of antibacterial ability in a mouse model

An experimental pneumonia mouse model was established by injecting *K. pneumoniae* Kp1049 intraperitoneally as previously reported (31). In brief, the bacteria were cultured in LB media to obtain a mixture with an OD_600_ of 0.5. The bacterial cultures were subsequently centrifuged (8,500 × *g*, 5 min) and washed twice with saline. Finally, the bacteria were resuspended to 1 × 10^11^ CFU/mL to determine the survival rate of the mice, and 1 × 10^10^ CFU/mL bacteria were used to quantify the bacterial loads. One hundred microliters of bacterial suspension was injected into the peritoneal cavity of mice to establish a bacteremia infection model. The phage solutions were prepared with different MOIs and were injected intraperitoneally 1 h after bacterial infection, with saline injection serving as the negative control. For mouse survival, 30 female C57BL/6 N mice were intraperitoneally injected with bacteria for infection and divided into 6 groups (*n* = 5). The survival rates of the mice infected with bacteria were monitored and recorded for 7 days. For bacterial loads, 12 female C57BL/6 N mice were intraperitoneally injected with bacteria for infection and divided into 3 groups (*n* = 6). The organs of the mice (heart, liver, spleen, lung, and kidney) were collected separately after 48 h of treatment, and the bacterial loads were analyzed via colony formation experiments (CFU/g) conducted on 1.5% LB agar plates to demonstrate the therapeutic effect of phage vB_KpnP_Henu1_3 on pneumonia model mice.

### Statistical analysis

All experiments were performed with at least three independent biological replicates. Data are presented as the mean ± standard deviation (SD) unless otherwise indicated. Statistical significance was defined as *P < 0.05, **P < 0.01, and ***P < 0.001 for all analyses. Data processing and visualization were conducted using GraphPad Prism 9.0 (GraphPad Software, Inc., Boston, MA, USA).

## RESULTS

### Isolation and morphology of phage vB_KpnP_Henu1_3

**Fig 1:**
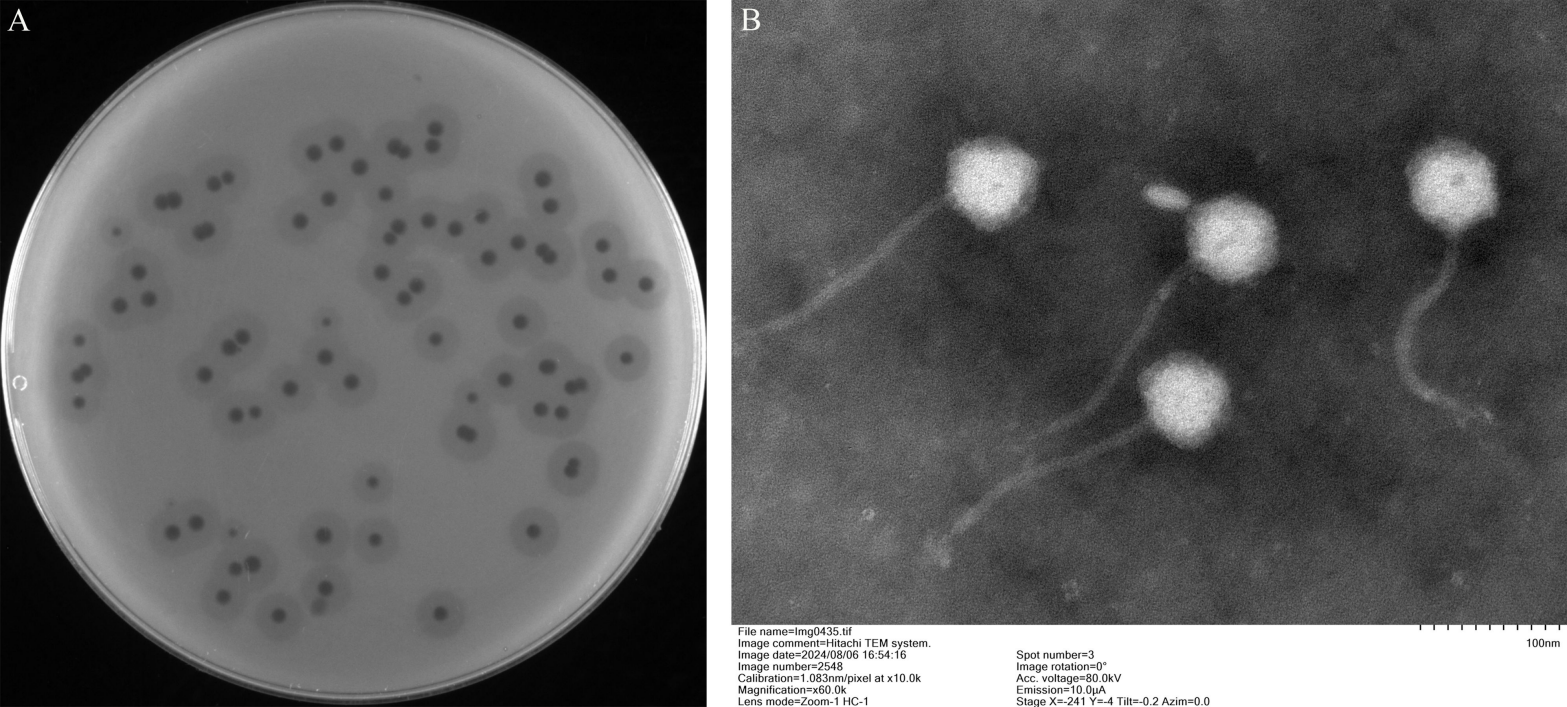
Morphological observation of phage vB_KpnP_Henu1_3. (**A**) Phage plaques formed on the lawn of *K. pneumoniae* Kp1049 at 37°C for 12 h. (**B**) TEM of phage vB_KpnP_Henu1_3.

The phage was isolated from hospital sewage using the clinical *K. pneumoniae* Kp1049 as a host. The filtered effluent was applied to a lawn containing the *K. pneumoniae* Kp1049. After five rounds of successive picking of individual phage plaques, homogeneous, clear plaques with halos were observed following 12 h of incubation at 37°C (Fig. 1A). TEM analysis revealed that vB_KpnP_Henu1_3 has an icosahedral head with an average diameter of 69.13 ± 0.67 nm and a noncontractile long tail averaging 179.86 ± 2.01 nm in length (Fig. 1B).

### Host range of phage vB_KpnP_Henu1_3

Phages are strictly host-selective, so the host range of phages determines their clinical applications. *K. pneumoniae* Kp1049, the host bacterium of phage vB_KpnP_Henu1_3, is a K1-type *K. pneumoniae* strain. To test the host range of phage vB_KpnP_Henu1_3, 30 clinical isolates of *K. pneumoniae* and other gram-negative bacteria were employed for the detection of phage vB_KpnP_Henu1_3 infectivity. The K-types of *K. pneumoniae* were identified via *wzi* gene sequencing (32). The results demonstrate that phage vB_KpnP_Henu1_3 infected all the tested K1-type *K. pneumoniae* strains but failed to infect other capsular types of *K. pneumoniae*, *E. coli*, *A. baumannii,* or *P. aeruginosa* (Table 1). Furthermore, the phage vB_KpnP_Henu1_3 exhibited an EOP exceeding 70% against all tested clinical isolates of K1-type *K. pneumoniae* (Table 1). These results indicated that vB_KpnP_Henu1_3 is a *Klebsiella* phage with strict host restriction.

**TABLE 1:** Host range analysis of phage vB_KpnP_Henu1_3 against 30 strains^*a*^

Species	Strains	Capsular type	Susceptibility	Origin	EOP^*b*^
*K. pneumoniae*	Kp1049	K1	++++	The First Affiliated Hospital of Henan University	100
	Kp0311	K1	++++	The First Affiliated Hospital of Henan University	92
	Kp0822	K1	++++	The First Affiliated Hospital of Henan University	89
	Kp407	K1	++++	Henan Provincial People’s Hospital	76
	Kp0918	K1	++++	The First Affiliated Hospital of Henan University	83
	Kp408	K2	–	Henan Provincial People’s Hospital	0
	Kp1203	K2	–	The First Affiliated Hospital of Henan University	0
	Kp1616	K2	–	The First Affiliated Hospital of Henan University	0
	Kp0524	K2	–	The First Affiliated Hospital of Henan University	0
	Kp1904-2431	K19	–	The First Affiliated Hospital of Henan University	0
	Kp302	K19	–	Henan Provincial People’s Hospital	0
	Kp309	K63	–	Henan Provincial People’s Hospital	0
	Kp403	K62	–	Henan Provincial People’s Hospital	0
	Kp2640	K28	–	The First Affiliated Hospital of Henan University	0
	Kp126N	K28	–	The First Affiliated Hospital of Henan University	0
	Kp306N	K14,K64	–	The First Affiliated Hospital of Henan University	0
	Kp347N	K14,K64	–	The First Affiliated Hospital of Henan University	0
	Kp2001-0185	K14,K64	–	Henan Provincial People’s Hospital	0
	Kp2001-0219	K14,K64	–	Henan Provincial People’s Hospital	0
	Kp2011-3676	K14,K64	–	Henan Provincial People’s Hospital	0
	Kp1901-0124	K14,K64	–	Henan Provincial People’s Hospital	0
	Kp120804	K62	–	Henan Provincial People’s Hospital	0
	Kp120819	K63	–	Henan Provincial People’s Hospital	0
	Kp57N	K63	–	The First Affiliated Hospital of Henan University	0
	Kp0953	K19	–	The First Affiliated Hospital of Henan University	0
	Kp0706	K14	–	The First Affiliated Hospital of Henan University	0
	Kp2828	K16	–	The First Affiliated Hospital of Henan University	0
*E. coli*	Ec1033		–	The First Affiliated Hospital of Henan University	0
*A. baumannii*	Ab2055		–	The First Affiliated Hospital of Henan University	0
*P. aeruginosa*	Pa3046		–	The First Affiliated Hospital of Henan University	0

^*a*^ ++++, plaques at 10^6^; +++, plaques at 10^5^; ++, plaques at 10^4^; –, no plaques.

^*b*^ EOP is calculated in percent as the PFU/mL of the phages on the test strain divided by the PFU/mL obtained on strain Kp1049 multiplied by 100.

### Biological characterization of phage vB_KpnP_Henu1_3

**Fig 2:**
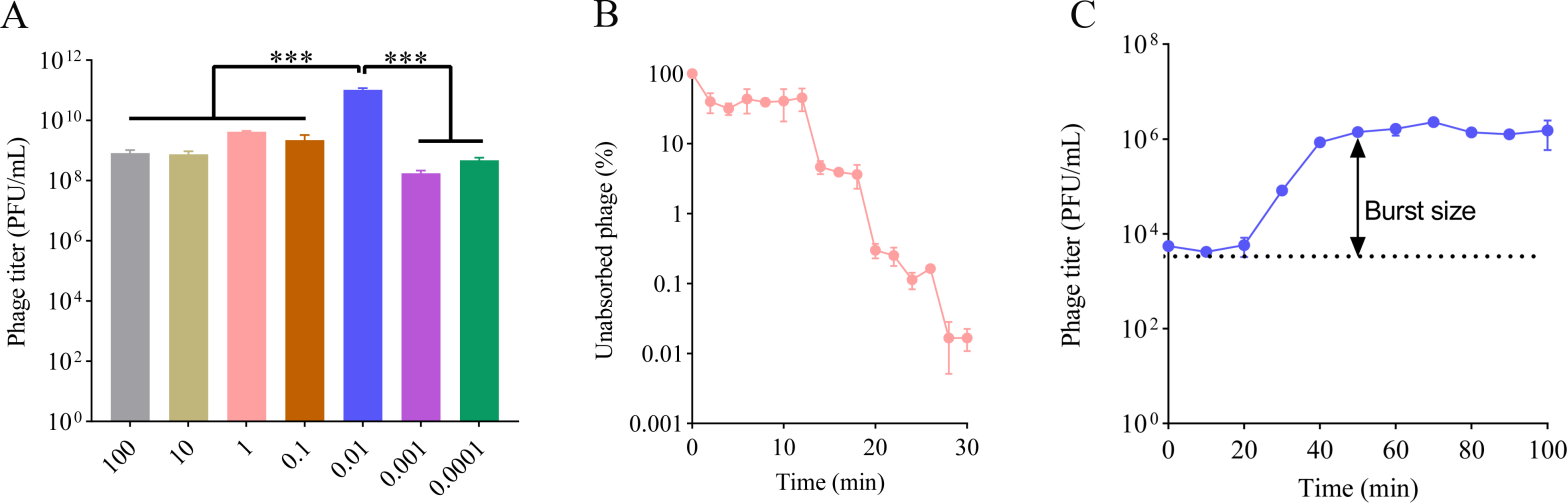
Biological characterization of phage vB_KpnP_Henu1_3. (**A**) Determination of the optimal MOI. Bacteria were infected with phage at various MOIs, adsorbed (37°C, 10 min), centrifuged to remove unadsorbed phages, incubated for replication (37°C, 2 h), and the progeny phage titer was determined by plaque assay. (**B**) Adsorption kinetics of phage vB_KpnP_Henu1_3 to *K. pneumoniae* Kp1049 at an MOI of 0.01, showing the percentage of unadsorbed phages over time as determined by quantifying free phage titers in the supernatant. (**C**) One-step growth curve analysis of phage vB_KpnP_Henu1_3, revealing its latent period and burst size, as determined by quantifying progeny phage titers at intervals following synchronous infection of *K. pneumoniae* Kp1049. Data represent means ± SD (*n*=3), statistical analyses were conducted using Student’s *t*-test for comparisons between groups. ***P < 0.001, **P < 0.01, *P < 0.05.

To determine the best MOI, phage vB_KpnP_Henu1_3 was mixed with the host *K. pneumoniae* Kp1049 at different MOIs. The titer was significantly greater than that of the other groups when *K. pneumoniae* Kp1049 was infected with vB_KpnP_Henu1_3 at an MOI of 0.01, indicating that 0.01 was the optimal MOI for phage vB_KpnP_Henu1_3 (Fig. 2A). Adsorption curve analysis revealed that more than 90% of phage vB_KpnP_Henu1_3 completely adsorbed to the host *K. pneumoniae* Kp1049 after 15 min of incubation (Fig. 2B). The one-step growth curve of phage vB_KpnP_Henu1_3 was plotted on the basis of the phage titer at different incubation times. The results showed that the latent period of phage vB_KpnP_Henu1_3 was 20 min, and the phage titer gradually increased and then plateaued at 50 min. The burst size of phage vB_KpnP_Henu1_3 is approximately 253 ± 54 PFU per infected cell (Fig. 2C).

### Tolerance of phage vB_KpnP_Henu1_3 to temperature and pH

**Fig 3:**
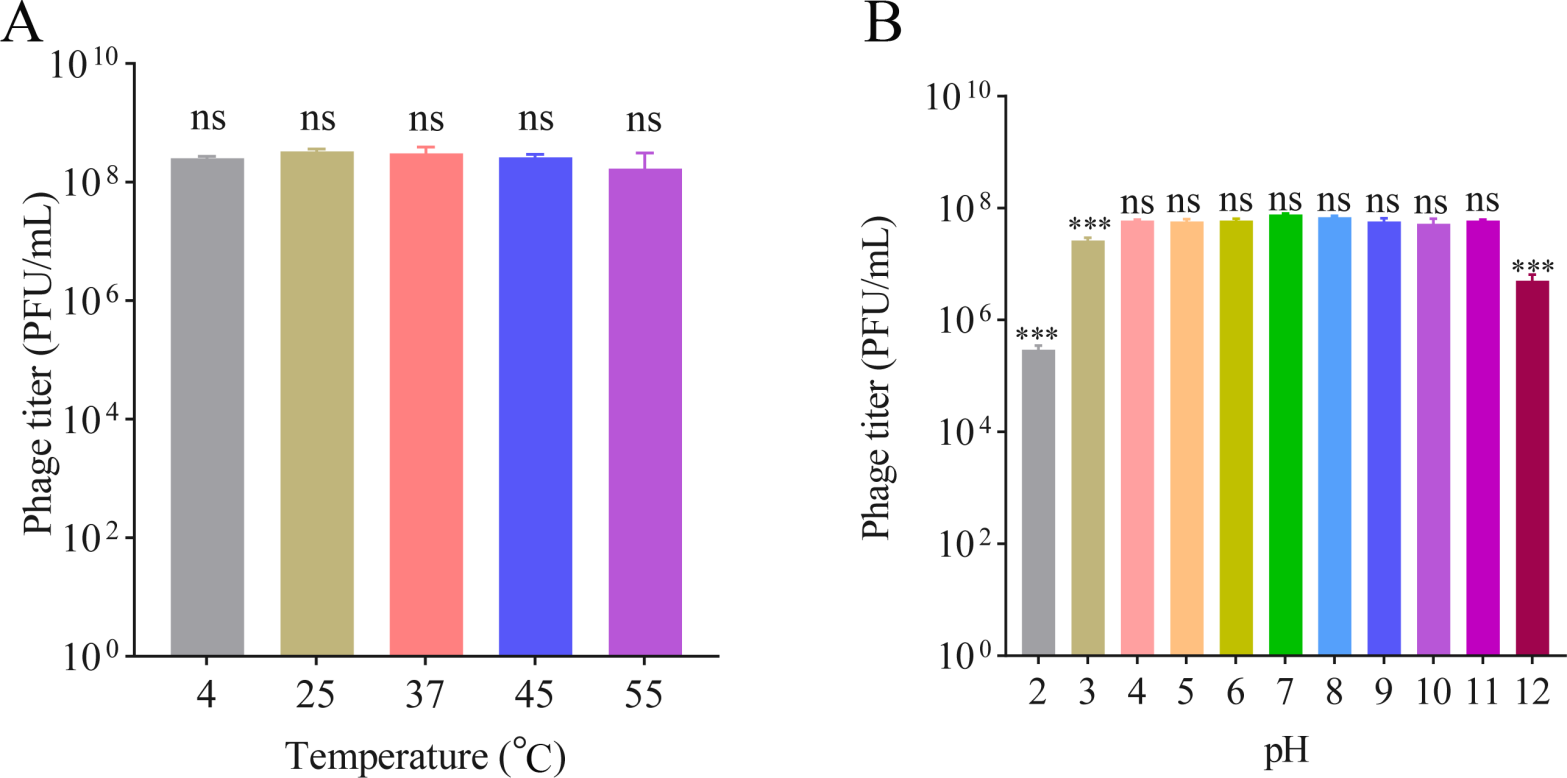
Sensitivity of phage vB_KpnP_Henu1_3 to temperature and pH. (**A**) Thermal stability of phage vB_KpnP_Henu1_3. The purified phage vB_KpnP_Henu1_3 suspensions were incubated at 4°C, 25°C, 37°C, 45°C, and 55°C for 12 h, after which the phage titer was detected. (**B**) pH stability of phage vB_KpnP_Henu1_3. vB_KpnP_Henu1_3 suspensions were incubated at different pH values ranging from 2 to 12 for 12 h, after which the phage titer was detected. Data are shown as the means ± SD (*n* = 3). Statistical differences among groups were analyzed using the Kruskal-Wallis test (a non-parametric alternative to ANOVA), followed by Dunn’s post hoc test for pairwise comparisons. ***P < 0.001, ns, not significant.

The infection stability of phages in different environments is critical for phage applications. We tested phage vB_KpnP_Henu1_3 infection in the host *K. pneumoniae* Kp1049 at different temperatures and pH values. The phage titer of vB_KpnP_Henu1_3 did not significantly change when the mixture was incubated at 4°C–55°C for 12 h (Fig. 3A). These data indicated that phage vB_KpnP_Henu1_3 could tolerate a wide range of temperatures and was suitable for use at temperatures less than 55°C. The phage vB_KpnP_Henu1_3 shows profound stability at pH values ranging from 3–11 for 12 h, while the phage titer significantly decreased at pH values below 3 or above 12 (Fig. 3B).

### The characterization of phage vB_KpnP_Henu1_3 genome

**Fig 4:**
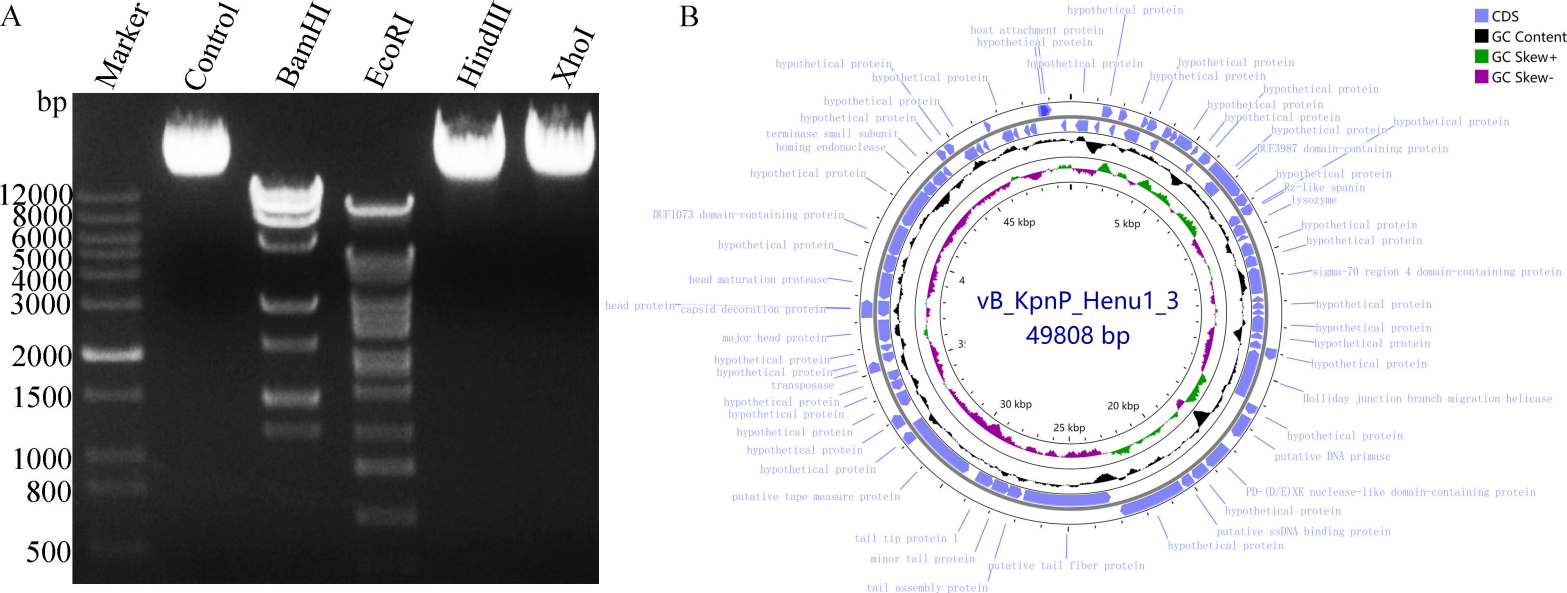
Genomic DNA and comprehensive genome map of phage vB_KpnP_Henu1_3. (**A**) Genomic DNA of phage vB_KpnP_Henu1_3 was subjected to restriction digestion by four restriction enzymes, namely, *Bam*HI, *Eco*RI, *Hin*dIII, and *Xho*I. (**B**) Complete genome map of vB_KpnP_Henu1_3, which consists of 49,808 base pairs. A genome map of vB_KpnP_Henu1_3 was generated via the Proksee GC viewer tool. ORFs encoding all genes, annotated genes, and the GC content are depicted.

**Fig 5:**
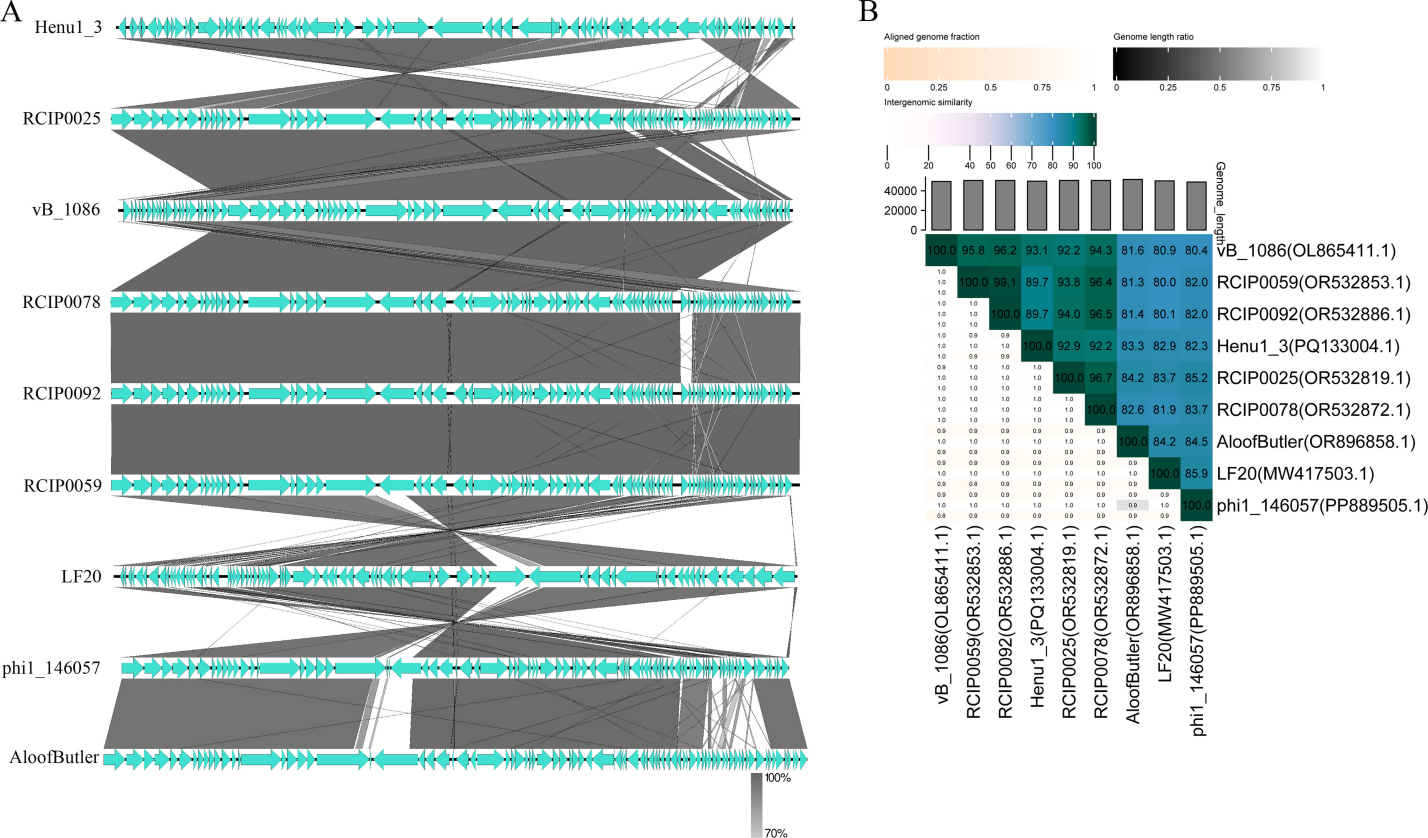
Compared genomic analysis of phage vB_KpnP_Henu1_3. (**A**) Alignment of vB_KpnP_Henu1_3 together with similar genomes of other phages. (**B**) Percentage sequence similarity between the phage vB_KpnP_Henu1_3 genome and homologous phages. The values were calculated via VIDIRIC.

The genome of phage vB_KpnP_Henu1_3 is a double-stranded DNA with a total length of 49,808 bp and a G + C content of 50.76%, which can be digested by *Bam*HI and *Eco*RI, but not *Hin*dIII or *Xho*I (Fig. 4A). The phage vB_KpnP_Henu1_3 genome contains 75 ORF (GenBank accession number: PQ133004.1), and no tRNA genes, known drug resistance genes, or virulence factor genes were identified (Fig. 4B). Only 18 of the 75 ORFs encoded by the vB_KpnP_Henu1_3 genome were annotated as functional proteins by BLASTp, and the rest of the ORFs were annotated as hypothetical proteins (Table 2). From the outside to the inside of phage vB_KpnP_Henu1_3, the genomic map is listed in the order of ORF feature, GC content, and GC skew (Fig. 4B). The BLAST analysis at the whole-genome level revealed that phage vB_KpnP_Henu1_3 is homologous to eight previously described *Klebsiella* phages (Table 3), and multiple sequence comparisons are shown in Fig. 5A. Phage vB_KpnP_Henu1_3 has the highest similarity with phage RCIP0025 at 92.9% (Fig. 5B). Based on ICTV guidelines, phage vB_KpnP_Henu1_3 was identified as a new species, along with RCIP0025, RCIP0059, and KOX1, in the genus *Webervirus* of the family *Drexlerviridae*.

**TABLE 2:** ORF analysis of the vB_KpnP_Henu1_3 phage genome

ORF	Strand	Start	Stop	Predicted protein function	Best-match BLASTp result	Query cover	E-values	Identity	Accession	MW (kDa)
1	−	180	725	Hypothetical protein	*Klebsiella* phage RCIP0025	100%	1E-130	99.45%	WPJ50710.1	20.85
2	−	982	1,200	Putative membrane protein	*Klebsiella* phage vB_KpnS-VAC6	100%	1E-43	97.22%	QZE50691.1	8.24
3	+	1,232	1,639	Hypothetical protein	*Klebsiella* phage P528	100%	6E-91	94.81%	QPX75233.1	15.34
4	−	1,623	1,856	Hypothetical protein	No hit	–^*a*^	–	–	–	8.63
5	+	1,903	2,232	Hypothetical protein	*Klebsiella* phage RCIP0025	100%	5e-72	97.25%	WPJ50706.1	12.76
6	−	2,273	2,920	Hypothetical protein	*Klebsiella* phage KPP2020	53%	6E-44	66.38%	WCR32885.1	23.86
7	+	2,842	3,081	Hypothetical protein	*Klebsiella* phage vB_1086	100%	3E-52	100%	UJQ43207.1	8.76
8	+	3,082	3,459	Hypothetical protein	*Klebsiella* phage RCIP0078	100%	5E-81	98.33%	WPJ54583.1	14.28
9	−	3,469	3,780	Hypothetical protein	No hit					11.59
10	+	3,759	4,100	Hypothetical protein	*Klebsiella* phage vB_KpnS_IMGroot	100%	6E-78	99.12%	YP_009902522.1	12.41
11	+	4,093	4,332	Hypothetical protein	*Bacteriophage sp*.	100%	9E-52	100%	UVN04682.1	8.98
12	+	4,340	5,032	Hypothetical protein	*Klebsiella* phage vB_KpnD_Chell	100%	1E-170	98.70%	UGO54751.1	26.51
13	+	5,105	5,308	Membrane protein	*Klebsiella* phage MezzoGao	100%	7E-39	100%	YP_009792140.1	7.48
14	−	5,262	5,480	Hypothetical protein	*Klebsiella* phage KPP2020	76%	6E-22	80.00%	WCR32894.1	8.23
15	+	5,489	5,926	Hypothetical protein	*Klebsiella* phage vB_1086	100%	2e-100	100%	UJQ43199.1	15.82
16	+	6,051	7,619	DNA helicase	*Klebsiella* phage AloofButler	100%	0.0	99.62%	WWD15272.1	57.83
17	−	6,386	6,994	Hypothetical protein	*Klebsiella* phage KPP2020	100%	1e-110	87.13%	WCR32897.1	21.87
18	+	7,623	8,084	Hypothetical protein	*Klebsiella* phage vB_LZ2044	100%	1e-110	100%	WCF59171.1	17.75
19	+	8,096	8,500	Hypothetical protein	No hit					15.34
20	−	8,153	8,578	Rz-like spanin	*Klebsiella* phage vB_KpnS_KpV522	100%	1e-96	100%	YP_009787676.1	14.88
21	−	8,575	9,057	Lysozyme	*Klebsiella* phage vB_1086	100%	3e-115	99.38%	UJQ43195.1	17.93
22	−	9,059	9,274	Hypothetical protein	*Klebsiella* phage vB_1086	100%	4e-41	100%	UJQ43194.1	7.56
23	−	9,409	9,981	Hypothetical protein	*Klebsiella* phage vB_LZ2044	100%	1e-137	100%	WCF59170.1	21.67
24	−	9,978	10,469	Hypothetical protein	*Klebsiella* phage RCIP0025	100%	3e-115	99.39%	WPJ50688.1	18.50
25	−	10,508	11,638	DNA repair exonuclease	*Klebsiella* phage vB_KpnS_KpV522	100%	0.0	99.47%	YP_009787671.1	42.45
26	−	11,734	11,982	Hypothetical protein	*Klebsiella* phage PKP126	100%	1e-52	100%	YP_009284873.1	9.44
27	−	11,982	12,272	Hypothetical protein	*Bacteriophage sp*.	100%	7e-65	100%	UVN04668.1	10.62
28	−	12,283	12,519	Hypothetical protein	Klebsiella pneumoniae	100%	6e-50	100%	WP_216264657.1	9.07
29	−	12,523	13,254	Hypothetical protein	*Klebsiella* phage vB_1086	100%	0.0	99.59%	UJQ43187.1	27.85
30	−	13,257	13,535	Hypothetical protein	*Klebsiella* phage vB_1086	100%	4e-59	100%	UJQ43186.1	10.07
31	−	13,610	13,939	Hypothetical protein	*Klebsiella* phage RCIP0059	100%	6e-74	100%	WPJ53215.1	12.14
32	+	13,838	14,290	Hypothetical protein	*Klebsiella* phage vB_KleS-HSE3	84%	0.013	38.06%	QIN95008.1	17.04
33	−	14,014	16,050	DNA helicase	*Klebsiella* phage vB_Kpn_K31PH164	100%	0.0	99.85%	CAK6589201.1	77.10
34	+	16,141	16,542	Hypothetical protein	*Enterobacteriaceae*	100%	7e-93	100%	WP_216264666.1	15.24
35	+	16,654	17,580	Putative DNA primase	*Klebsiella* phage vB_LZ2044	100%	0.0	100%	WCF59221.1	34.71
36	+	18,077	19,123	Exonuclease	*Klebsiella* phage 209	100%	0.0	98.56%	WKV32679.1	39.34
37	+	19,183	19,839	Hypothetical protein	*Klebsiella* phage vB_1086	100%	8e-161	100%	UJQ43180.1	24.34
38	+	19,876	20,343	Putative ssDNA-binding protein	*Klebsiella* phage PWKp17	100%	3e-110	98.71%	UJD05649.1	17.57
39	+	20,392	22,974	Tail fiber protein	*Klebsiella* phage P287	98%	0.0	98.82%	XDJ01954.1	91.29
40	−	23,198	26,896	Putative tail fiber protein	*Klebsiella* phage vB_LZ2044	100%	0.0	99.92%	WCF59234.1	136.69
41	−	26,984	27,586	Tail assembly protein	*Klebsiella pneumoniae*	100%	2e-140	100%	WP_216264494.1	20.72
42	−	27,561	28,298	Minor tail protein	*Klebsiella* phage vB_KpnS_IMGroot	100%	0.0	100%	YP_009902490.1	28.42
43	−	28,300	29,052	Tail tip protein L	*Klebsiella* phage RCIP0040	100%	0.0	100%	WPJ51778.1	27.52
44	−	29,469	32,534	Putative tape measure protein	*Klebsiella* phage vB_LZ2044	100%	0.0	98.92%	WCF59235.1	110.20
45	+	31,948	32,409	Hypothetical protein	*Klebsiella* phage mfs	96%	1e-88	95.92%	UYE93665.1	15.96
46	+	32,668	33,213	Hypothetical protein	*Klebsiella* phage KPP2020	33%	8e-15	63.93%	WCR32835.1	20.60
47	−	33,275	33,931	Hypothetical protein	*Klebsiella* phage vB_1086	100%	8e-160	99.54%	UJQ43170.1	24.20
48	−	34,025	34,456	Hypothetical protein	Stenotrophomonas phage vB_SmeS_BUCT705	100%	8e-102	100%	UNY50352.1	15.80
49	−	34,446	34,883	HK97 gp10 family phage protein	*Klebsiella pneumoniae*	100%	8e-103	100%	WP_216264529.1	16.02
50	+	35,014	35,454	Transposase	*Klebsiella* phage KPP2020	100%	3e-87	86.99%	WCR32841.1	16.33
51	−	35,259	35,675	Hypothetical protein	*Klebsiella* phage RCIP0085	100%	3e-97	100%	WPJ55022.1	15.44
52	−	35,729	36,025	Hypothetical protein	*Klebsiella* phage BUCT556A	100%	2e-62	98.98%	UPT53762.1	11.22
53	−	36,118	37,077	Major head protein	*Klebsiella* phage GML-KpCol1	100%	0.0	99.69%	YP_009796926.1	35.23
54	+	37,166	37,876	Capsid decoration protein	Klebsiella phage vB_KpnS-VAC11	69%	2e-72	70.91%	QZE51044.1	25.73
55	−	37,193	37,870	Head protein	Klebsiella phage Sanco	99%	8e-131	87.50%	QBZ71160.2	22.93
56	−	37,922	39,052	Putative major capsid protein	*Klebsiella* phage LAPAZ	100%	0.0	99.47%	XAG95214.1	41.08
57	−	39,049	39,819	Hypothetical protein	*Klebsiella* phage vB_1086	100%	0.0	100%	UJQ43161.1	29.34
58	−	39,809	41,128	DUF1073 domain-containing protein	*Stenotrophomonas* phage vB_SmeS_BUCT705	100%	0.0	100%	UNY50343.1	48.54
59	−	41,175	42,794	Hypothetical protein	*Klebsiella* phage RCIP0025	98%	0.0	99.81%	WPJ50655.1	61.96
60	−	42,769	43,320	Homing endonuclease	*Klebsiella* phage vB_LZ2044	92%	1e-122	99.41%	WCF59217.1	20.35
61	−	43,336	43,944	Terminase small subunit	*Klebsiella* phage vB_Kpl_K53PH164C2	100%	3e-144	97.03%	CAK6604553.1	22.44
62	−	43,941	44,204	Hypothetical protein	Klebsiella phage PKP126	100%	3e-57	100%	YP_009284915.1	9.92
63	+	44,240	44,566	Hypothetical protein	Escherichia coli	73%	2e-50	100%	WP_236527818.1	12.31
64	+	44,620	44,967	Hypothetical protein	No hit					13.29
65	−	45,049	45,624	Hypothetical protein	*Klebsiella* phage vB_1086	100%	2e-139	98.95%	UJQ43152.1	22.10
66	−	45,621	45,893	Hypothetical protein	*Klebsiella* phage vB_1086	100%	4e-60	100%	UJQ43151.1	10.25
67	−	45,957	46,169	Hypothetical protein	*Klebsiella* phage vB_1086	100%	7e-45	98.57%	UJQ43150.1	8.36
68	+	46,390	46,611	Hypothetical protein	*Escherichia coli*	76%	4e-26	89.29%	WP_236273106.1	8.41
69	−	46,831	47,319	Hypothetical protein	*Klebsiella* phage vB_1086	100%	2e-114	98.15%	UJQ43147.1	18.58
70	−	47,319	47,537	Hypothetical protein	*Klebsiella* phage Shelby	100%	1e-41	97.22%	YP_009903372.1	8.22
71	−	47,799	48,044	Hypothetical protein	*Klebsiella* phage KPN N141	100%	2e-53	100%	YP_009791613.1	9.29
72	−	48,044	48,346	Hypothetical protein	*Klebsiella* phage vB_1086	100%	1e-67	99.00%	UJQ43143.1	11.50
73	+	48,539	48,949	Hypothetical protein	*Klebsiella* phage KPP2020	90%	1e-70	84.55%	WCR32871.1	14.92
74	+	48,654	49,064	Host attachment protein	*Klebsiella* phage KL	65%	5e-08	42.70%	YP_009902841.1	15.92
75	−	49,390	49,614	Hypothetical protein	*Klebsiella* phage vB_1086	100%	1e-49	98.65%	UJQ43139.2	8.68

^*a*^ –, no data.

**TABLE 3:** Comparison of vB_KpnP_Henu1_3 and homologous bacteriophages

Phage name	Genome size	Type	Query cover of vB_KpnP_Henu1_3	Identity of vB_KpnP_Henu1_3	Accession
*Klebsiella* phage Henu1_3	49,808 bp	Circular	100%	100%	PQ133004
*Klebsiella* phage RCIP0025	50,503 bp	Linear	96%	98.14%	OR532819.1
*Klebsiella* phage vB_1086	49,473 bp	Linear	93%	98.24%	OL865411.1
*Klebsiella* phage RCIP0078	50,552bp	Linear	94%	98.08%	OR532872.1
*Klebsiella* phage RCIP0092	50,486 bp	Linear	92%	97.71%	OR532886.1
*Klebsiella* phage RCIP0059	50,487 bp	Linear	92%	97.63%	OR532853.1
*Klebsiella* phage LF20	50,107 bp	Linear	86%	96.31%	MW417503.1
*Klebsiella* phage phi1_146057	48,907 bp	Circular	85%	96.23%	PP889505.1
*Klebsiella* phage AloofButler	51,584 bp	Linear	88%	96.15%	OR896858.1

### Evaluation of phage vB_KpnP_Henu1_3 against *K. pneumoniae* Kp1049 *in vitro*

**Fig 6:**
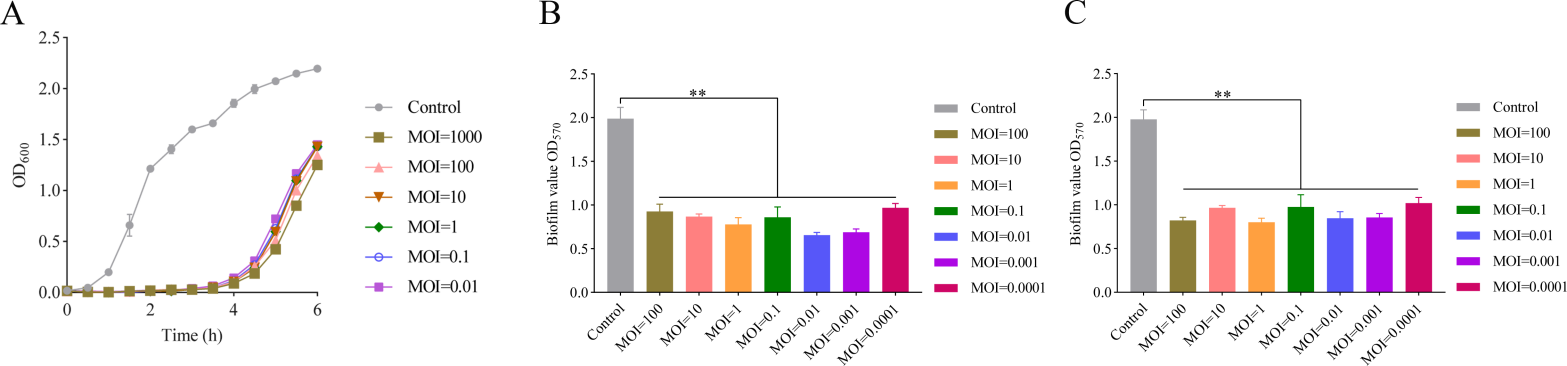
Assessment of the efficacy of phage vB_KpnP_Henu1_3 against *K. pneumoniae* Kp1049 *in vitro*. (**A**) Inhibition of *K. pneumoniae* Kp1049 growth by phage vB_KpnP_Henu1_3. *K. pneumoniae* Kp1049 was cocultivated with vB_KpnP_Henu1_3 at different MOIs at 37°C with shaking for 6 h, and the absorbance (OD_600_) of each group was measured every 0.5 h. (**B**) The biofilm inhibition effect of phage vB_KpnP_Henu1_3. *K. pneumoniae* kp1049 was coincubated with phage vB_KpnP_Henu1_3 at MOIs of 0.0001, 0.001, 0.01, 0.1, 1, 10, and 100 for 12 h at 37°C. After incubation, the biofilm remaining after phage vB_KpnP_Henu1_3 treatment was measured via a CV assay. (**C**) The biofilm disruption effect of phage vB_KpnP_Henu1_3. *K. pneumoniae* Kp1049 was incubated at 37°C for 24 h to form a mature biofilm, and then phage vB_KpnP_Henu1_3 at MOIs of 0.0001, 0.001, 0.01, 0.1, 1, 10, and 100 was coincubated with the formed biofilm for another 12 h at 37°C. The remaining biofilm was detected via a CV assay. The data are shown as the means ± SD (*n*=3). Statistical significance was analyzed by Kruskal-Wallis test, a non-parametric alternative to one-way ANOVA, followed by Dunn’s post hoc test for pairwise comparisons (***P < 0.001, **P < 0.01, *P < 0.05).

The strong lytic activity of phages is the basis for phage therapy used in the clinic. In this study, the lytic activity of phage vB_KpnP_Henu1_3 was measured by mixing it with *K. pneumoniae* Kp1049 in the pre-logarithmic growth phase at different MOIs and incubating it for 6 h. The growth curves revealed that treatment with phage vB_KpnP_Henu1_3 at MOIs ranging from 0.01 to 1,000 completely inhibited *K. pneumoniae* Kp1049 within 4 h (Fig. 6A). The OD_600_ of each culture group gradually increased over time, suggesting that *K. pneumoniae* Kp1049 may have developed phage resistance through mutations.

Biofilms are one of the means by which bacteria defend themselves against harsh external environments and are highly resistant to antimicrobial agents. Biofilm formation can exacerbate infections in patients, resulting in harsh treatment and causing great concern in the healthcare environment. Biofilm formation by *K. pneumoniae* can facilitate colonization in multiple anatomical sites, including the gastrointestinal, respiratory, and urogenital tracts, and may subsequently promote the development of invasive infections (33). Several studies have reported that phage or phage-derived proteins are effective at removing biofilms (34, 35). Therefore, the ability of phages to inhibit biofilm formation or remove mature biofilms is among the important evaluation indices of phages used for therapy. In this study, we evaluated the inhibition and removal of biofilms by phage vB_KpnP_Henu1_3. Coincubation of *K. pneumoniae* Kp1049 with phage vB_KpnP_Henu1_3 at varying MOIs (0.0001–100) significantly reduced biofilm biomass (Fig. 6B). Mature biofilms were also significantly disrupted when the mature biofilms were incubated with phage vB_KpnP_Henu1_3 at MOIs ranging from 0.0001 to 100 (Fig. 6C). These results indicated that phage vB_KpnP_Henu1_3 could significantly inhibit biofilm formation and disrupt mature biofilms of *K. pneumoniae* Kp1049.

### Evaluation of phage vB_KpnP_Henu1_3 against *K. pneumoniae* Kp1049 *in vivo*

**Fig 7:**
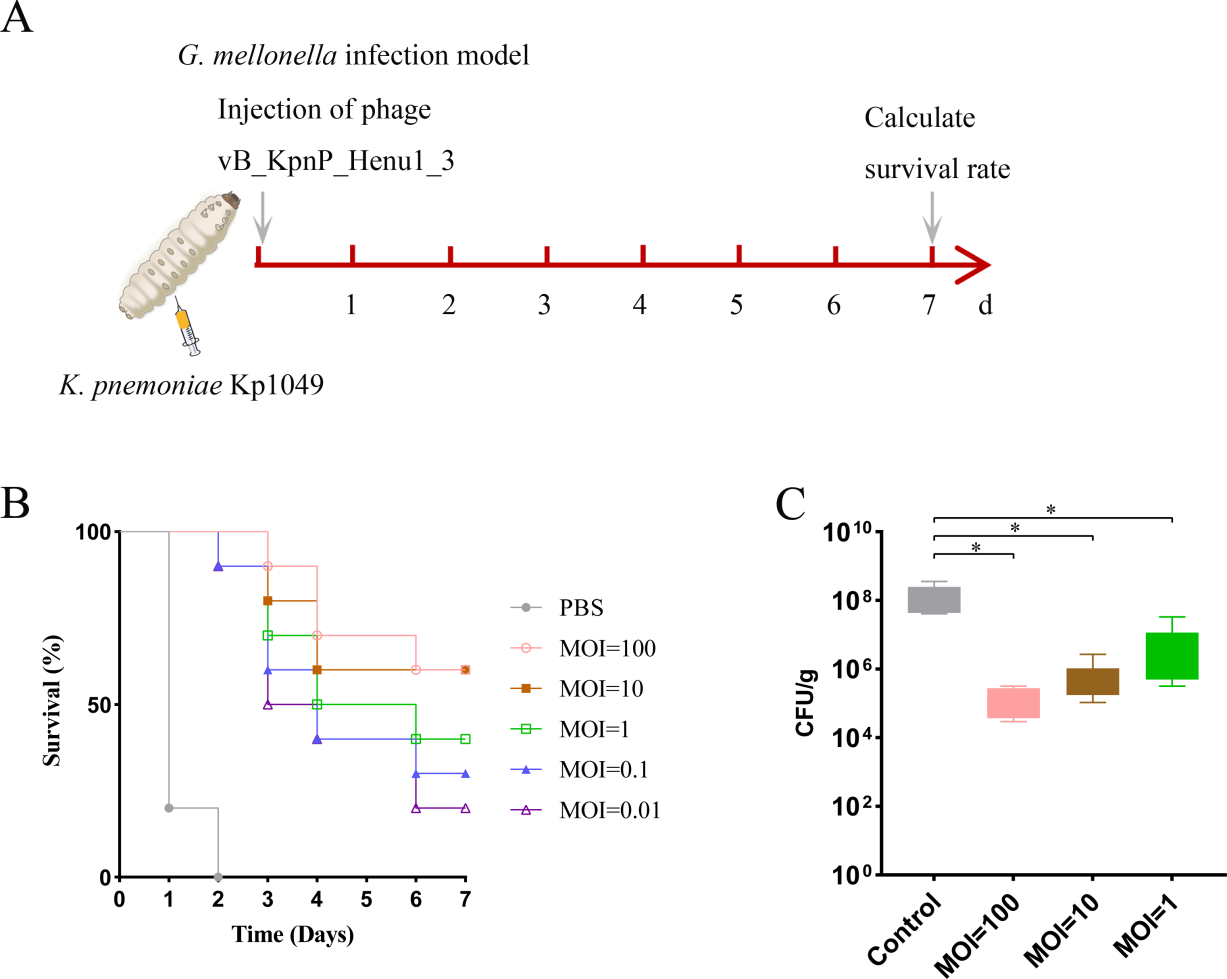
Phage therapy in the *G. mellonella* larvae model. (**A**) Schematic representation of phage vB_KpnP_Henu1_3 treating *K. pneumoniae* Kp1049-infected *G. mellonella* larvae. (**B**) Survival curves of *G. mellonella* (*n* = 10) infected with *K. pneumoniae* Kp1049 followed by phage vB_KpnP_Henu1_3 treatment at different MOIs. (**C**) Bacterial loads in infected *G. mellonella* larvae. *G. mellonella* larvae (*n* = 6) infected with *K. pneumoniae* Kp1049 were treated with phage vB_KpnP_Henu1_3 at MOIs of 1, 10, and 100. After treatment for 24 h, the bacterial loads were determined via the serial dilution method. Statistical significance was determined by Student’s *t*-test (*P < 0.05, vs. infected control).

The larvae of *G. mellonella*, an animal model prone to infection by a wide range of pathogenic microorganisms, have become an attractive model for the preliminary evaluation of virulence and efficacy (36). Exploring the antimicrobial effects of phages in *G. mellonella* larvae infection models has also become one of the means of initially evaluating the therapeutic efficacy of phages *in vivo* (37, 38). In this study, we evaluated the effect of phage vB_KpnP_Henu1_3 *in vivo* on *G. mellonella* larvae infected with *K. pneumoniae* Kp1049. Each *G. mellonella* larva was infected with *K. pneumoniae* Kp1049 at a concentration of 1 × 10^9^ CFU/mL and treated with phage vB_KpnP_Henu1_3 at MOIs of 0.01, 0.1, 1, 10, and 100 one hour later, with the PBS-treated group served as a negative control (Fig. 7A). The *G. mellonella* larvae treated with PBS died within 2 days, and the survival rates of *G. mellonella* larvae treated with phage vB_KpnP_Henu1_3 at MOIs of 0.01, 0.1, 1, 10, and 100 improved by 20%, 30%, 40%, 60%, and 60%, respectively (Fig. 7B). In addition, the loads of bacteria in *G. mellonella* larvae were significantly diminished in the phage vB_KpnP_Henu1_3 treatment group (Fig. 7C). The above results demonstrated that phage vB_KpnP_Henu1_3 significantly reduced the bacterial load of *K. pneumoniae* Kp1049 in infected *G. mellonella* larvae within 24 h.

**Fig 8:**
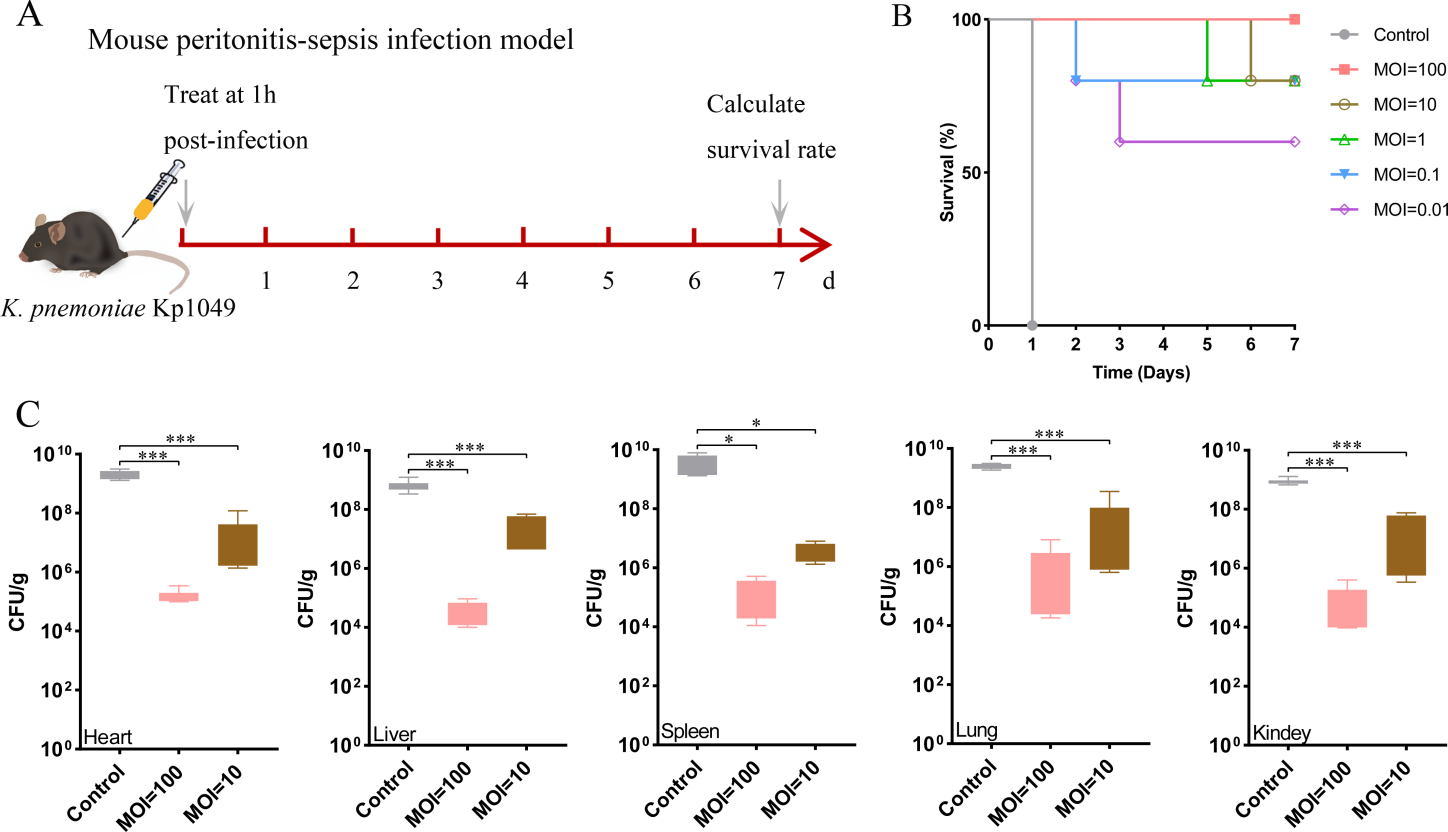
Phage therapy in a mouse bacteremia model. (**A**) Schematic representation of phage vB_KpnP_Henu1_3 treatment of *K. pneumoniae* Kp1049-infected mice. (**B**) Survival rate analysis of infected mice. Mice were infected with *K. pneumoniae* Kp1049 at a CFU/mL of 1 × 10^11^ and received phage therapy at different MOIs (100, 10, 1, 0.1, and 0.01) (*n* = 5). The survival of the mice was observed and recorded for 7 days. (**C**) Bacterial loads of different organs in a mouse bacteremia model. Mice were infected with *K. pneumoniae* Kp1049 at a concentration of 1 × 10^10^ CFU/mL via intraperitoneal injection (*n* = 4). After 1 h, the infected mice were treated with vB_KpnP_Henu1_3 at different MOIs. The bacterial loads were determined by counting the CFUs in the dissected and ground tissues. Statistical significance was determined by Student’s *t*-test (***P < 0.001, **P < 0.01, *P < 0.05, vs. untreated controls).

Furthermore, the therapeutic effect of phage vB_KpnP_Henu1_3 was evaluated via the peritoneal infection of mice with *K. pneumoniae* Kp1049. The mouse peritonitis-sepsis infection model was constructed by injection of 100 μL of *K. pneumoniae* Kp1049 (1 × 10^11^ CFU/mL). The same volume of phage vB_KpnP_Henu1_3 (MOI = 100, 10, 1, 0.1, 0.01) was used for treatment at 1 h post-infection, and the group in which the same volume of PBS was injected served as a negative control (Fig. 8A). The results revealed that all the mice in the negative control group died within 1 day, whereas the survival rates of the mice in the different MOI phage treatment groups significantly improved (Fig. 8B). At an MOI of 100, the survival rates of the mice even reached 100% (Fig. 8B). To evaluate the lytic efficiency of phage vB_KpnP_Henu1_3 against *K. pneumoniae* Kp1049, we measured the bacterial loads in various organs of the mice. The mice were injected with 100 μL of *K. pneumoniae* Kp1049 (1 × 10^10^ CFU/mL), and phage vB_KpnP_Henu1_3 (MOI = 100, 10) was used for treatment at 1 h post-infection. The organs were used for CFU counting after 24 h. The results confirmed that the CFUs/g of the heart, liver, spleen, lung, and kidney were all prominently reduced at MOIs = 100 or 10 (Fig. 8C). These results indicated that phage vB_KpnP_Henu1_3 could effectively lyse K1-type *K. pneumoniae in vivo* and improve the survival of the mice.

## DISCUSSION

*K. pneumoniae* is an important opportunistic pathogen that can cause a variety of diseases, such as respiratory tract, circulatory system, and wound infections, and is notorious for its high mortality (39). Owing to the production of β-lactamases, the prevalence of β-lactam resistance in *K. pneumoniae* has gradually increased, and the resistance to fluoroquinolones and carbapenems has also been reported (40). In addition, *K. pneumoniae* carbapenemase (KPC) mutations, which can reduce the efficacy of ceftazidime-avibactam (CZA), the pioneer antimicrobial agent for carbapenem-resistant Enterobacteriaceae infections, have been reported in hundreds of species (41). *K. pneumoniae* can be classified into two types: classical *K. pneumoniae* (cKP) and hypervirulent *K. pneumoniae* (hvKP). cKP isolates are highly diverse and important causes of nosocomial infections. HvKP is a common pathogen that causes pyogenic liver abscesses in young healthy people and was first identified in Taiwan in 1986 (42). HvKP can infect almost all parts of the body and cause serious infections, such as meningitis, endophthalmitis, pneumonia, bacteremia, liver abscesses, and skin tissue necrosis. Compared with cKP infections, hvKP infections have a higher mortality rate, a more rapid onset of disease, and a poorer prognosis, which makes postinfectious treatment of hvKP more difficult (43). On the basis of the available statistics of *K. pneumoniae* serotypes, hvKPs include mainly K1, K2, K5, and K57, of which K1 and K2 are the most common, accounting for approximately 70% of hvKPs (44, 45). The emergence of highly resistant and hypervirulent *K. pneumoniae* poses a great threat to the clinical treatment and control of *K. pneumoniae* infections (46, 47). The biofilm formation rate of hvKP was significantly higher than that of cKP (48). The hvKP strains not only formed denser and more cohesive biofilms but also exhibited more complex extracellular matrix (48, 49). Therefore, there is an urgent need to develop alternative therapies or new methods that can be combined with antibiotic therapy to address the great threat posed by antibiotic resistance. Phages, as one of the most promising means of addressing bacterial drug resistance, are also bound to play an important role in the treatment of *K. pneumoniae* infections. The mass isolation and characterization of *Klebsiella* phages will provide selective therapeutic options for the complex capsular polysaccharide (CPS) types associated with *K. pneumoniae* infection.

Lytic phages can lyse and eliminate their target bacteria and have unique advantages, such as high specificity, efficiency, multiplication rate, and resistance prevention ability (50). These properties increase their potential for combating antibiotic-resistant bacterial infections (51). Here, we isolated and reported a novel lytic phage, vB_KpnP_Henu1_3, from hospital sewage that exhibits interesting characteristics for feasible applications in controlling *K. pneumoniae*, specifically the K1 capsule type. The phage vB_KpnP_Henu1_3 was isolated by *K. pneumoniae* Kp1049 as the host bacterium and is capable of producing phage spots with halo rings (Fig. 1A). TEM revealed that phage vB_KpnP_Henu1_3 had an ortho-icosahedral head structure and a tail length of 179.86 ± 2.01 nm (Fig. 1B). Restriction-modification (RM) systems represent a ubiquitous and evolutionarily ancient defense mechanism in bacteria and archaea, serving as a primary barrier against bacteriophage infection (52). The double-stranded DNA phage vB_KpnP_Henu1_3, while susceptible to cleavage by multiple restriction endonucleases, demonstrates resistance to *Hin*dIII and *Xho*I digestion (Fig. 4A). Genomic analysis reveals the absence of recognition sites for *Hin*dIII and *Xho*I, and no DNA modification-related proteins (e.g., methyltransferases) are encoded in its genome. These findings suggest that vB_KpnP_Henu1_3 employs alternative strategies to circumvent host RM systems, independent of widespread DNA modifications. The genome length of phage vB_KpnP_Henu1_3 is 49,808 bp, and the G + C content is 50.76%. Most of the phages reported to date are double-stranded DNA, and a few are single-stranded DNA or RNA. The genome length and G + C content of phage vB_KpnP_Henu1_3 are consistent with those of most *Klebsiella* phages. The genome sequence of phage vB_KpnP_Henu1_3 shares the highest similarity of 98.14% and 96% coverage with that of *Klebsiella* phage RCIP0025. The genome of phage vB_KpnP_Henu1_3 is circular, whereas that of *Klebsiella* phage RCIP0025 is linear. However, there are no reports describing the relationships between circular or linear DNA and phage biological characteristics.

By testing the ability of phage vB_KpnP_Henu1_3 to infect 30 clinical isolates of *K. pneumoniae*, we found that phage vB_KpnP_Henu1_3 could solely target and lyse K1-type *K. pneumoniae*. Although both the K1 and K2 types of *K. pneumoniae* are highly virulent and viscous, the phage vB_KpnP_Henu1_3 failed to lyse clinical isolates of K2-type *K. pneumoniae*, including Kp408, Kp1203, Kp1616, and Kp0524. CPS is one of the crucial virulence factors of *K. pneumoniae* and the necessary receptor of most *Klebsiella* phages (53). Differences in the structure of the *K. pneumoniae* CPS between types K1 and K2 may be the main reason for the specificity of phage vB_KpnP_Henu1_3 infection (54). Correspondingly, depolymerase recognizes and degrades the bacterial CPS, thus enabling the phage to recognize and bind to secondary receptors (usually outer membrane proteins) on the bacterial surface (55). Phage depolymerases have been identified in many bacteriophages, which recognize and degrade CPS in a host-specific manner. The depolymerase Depo16 from phage ZK1 can specifically degrade the K1 serotype CPS of *K. pneumoniae* and increase the sensitivity of K1-type *K. pneumoniae* to the peritoneum macrophages (56). The depolymerase Dep42 derived from phage SH-KP152226, which is specific for the K47 capsule, was able to significantly inhibit biofilm formation or degrade formed biofilms (57). A capsule depolymerase specific for KL47-type CPS, which inhibited biofilms as well as the prevention and control of CRKP infections, was identified in the *Klebsiella* phage P560 genome (58). *Klebsiella* phage infection of fixed Capsular-type *K. pneumoniae* may be associated with phage-encoded depolymerases. In the genomes of phage vB_KpnP_Henu1_3, two ORFs are predicted to be putative tail fiber proteins that encode depolymerases (ORF39 and ORF40). Thus, these two genes may be among the main reasons for the selective infection of *K. pneumoniae* type K1 by phage vB_KpnP_Henu1_3.

The phage vB_KpnP_Henu1_3 acts in a lytic manner and can rapidly lyse K1-type *K. pneumoniae* with an optimal infection multiplicity of 0.01 (Fig. 2A). When *K. pneumoniae* Kp1049 was mixed with the phage vB_KpnP_Henu1_3, the phage was able to rapidly recognize *K. pneumoniae*, and the phage adsorbed to *K. pneumoniae* completely within 30 min (Fig. 2B). Phage vB_KpnP_Henu1_3 has a short incubation period of 20 min, and the burst size of phage vB_KpnP_Henu1_3 is approximately 253 ± 54 average progeny per infected cell (Fig. 2C). The lysis efficiency of phage vB_KpnP_Henu1_3 remains relatively low at 55°C, below pH 4 and above pH 11 (Fig. 3). The high burst size and lysis stability of phage vB_KpnP_Henu1_3 both set the stage for phage applications. Infection of *K. pneumoniae* Kp1049 with phage vB_KpnP_Henu1_3 at different MOIs delayed the entry of the bacteria into the log phase by approximately 4 h (Fig. 6A). However, after 4 h, *K. pneumoniae* rapidly entered the logarithmic phase, which was probably attributed to bacterial resistance to the phage. The generation of resistance during the incubation of phages with host bacteria *in vitro* is a common phenomenon observed in many studies (59, 60). The rapid development of bacterial resistance to phages is primarily caused by receptor mutations that reduce adsorption efficiency (61). Therefore, strategies such as developing phage cocktails targeting different receptors or combining phages with antibiotics can minimize the frequency of phage resistance emergence (62, 63). These approaches will help mitigate the risk of phage therapy failure due to resistance in the future. Further confirmation of a large reservoir of novel phages is a prerequisite for the clinical application of phages. Biofilms are one of the forms of bacteria that face extreme environments and are extremely resistant to antimicrobial agents. Numerous studies have shown that phages have a clear advantage in the formation and disruption of biofilms. The phage vB_KpnP_Henu1_3 not only inhibits planktonic bacteria but also inhibits the formation of bacterial biofilms and disrupts mature biofilms (Fig. 6B and C). Genome sequencing revealed that phage vB_KpnP_Henu1_3 does not encode virulence- or resistance-related proteins. However, the functions of most of the proteins encoded by phage vB_KpnP_Henu1_3 are unknown. Therefore, further studies to reveal the functions of proteins encoded by phage vB_KpnP_Henu1_3 will lay a solid foundation for the clinical application of phages.

The ability of phages to find pathogenic bacteria and lyse them in animals is the basis for their clinical application. Currently, animal models commonly used to evaluate phage therapy for *K. pneumoniae* infections include zebrafish, *G. mellonella* larvae, and mice (38, 64–66). To further evaluate the antimicrobial effect of phage vB_KpnP_Henu1_3 *in vivo*, two animal infection models were established in this study, including *G. mellonella* larvae and mice. The results indicated that phage vB_KpnP_Henu1_3 rapidly reduced the number of bacteria in the animal infection model, and the higher the MOI was, the lower the bacterial load (Fig. 7C and 8C). Bacteria can swiftly foster phage resistance *in vitro*, while a single administration of phage vB_KpnP_Henu1_3 resulted in a significant increase in animal survival (Fig. 7B and 8B). When the MOI was 100, the survival rate of the mice reached 100% (Fig. 8B). While phage vB_KpnP_Henu1_3 demonstrated an optimal MOI of 0.01 under *in vitro* conditions, *in vivo* efficacy required significantly higher phage titers. This observation aligns with previously reported phage behavior and likely reflects the physiological challenges of systemic infections (38). The requirement for elevated phage concentrations *in vivo* may be explained by several factors: (i) extensive bacterial dissemination throughout host tissues (ii), reduced phage penetration at infection sites due to biological barriers, and (iii) the need to overcome rapid bacterial replication in host microenvironments. These physiological constraints collectively necessitate higher phage loads to ensure adequate bacterial adsorption and subsequent lysis for therapeutic efficacy.

In this study, we successfully isolated and characterized a novel lytic phage that specifically targets K1-type hvKP. We carried out morphological observation, host range analysis, biological characterization, sensitivity analysis, genomic and evolutionary characterization, and these findings revealed that this phage exhibited excellent tolerance to a broad range of pH values and a wide temperature range. The phage vB_KpnP_Henu1_3 was effective at halting biofilm formation and disrupting mature biofilms, and no genes encoding virulence-, lysogenic-, integrase-, or antibiotic resistance-related genes were found in the genome. In addition, vB_KpnP_Henu1_3 showed promising antibacterial effects *in vivo* and *in vitro*, indicating great potential as a promising alternative for antimicrobial therapy.

## Supplementary Material

Reviewer comments

## Data Availability

The genome sequence and annotation of phage vB_KpnP_Henu1_3 have been submitted to the GenBank database under accession number PQ133004.1. The raw sequencing data have been deposited in the National Microbiology Data Center under BioProject accession number NMDC10020201.

## SUPPLEMENTAL MATERIAL

Tables S1 spectrum.00931-25-s0001.doc The isolation information of clinical isolates used in this study.

OPEN PEER REVIEW reviewer-comments.pdf An accounting of the reviewer comments and feedback.

ASM does not own the copyrights to Supplemental Material that may be linked to, or accessed through, an article. The authors have granted ASM a non-exclusive, world-wide license to publish the Supplemental Material files. Please contact the corresponding author directly for reuse.

## References

[1] Walker KA, Miller VL. 2020. The intersection of capsule gene expression, hypermucoviscosity and hypervirulence in Klebsiella pneumoniae. Curr Opin Microbiol 54:95–102. doi:10.1016/j.mib.2020.01.006 32062153 PMC8121214

[2] Pu D, Zhao J, Chang K, Zhuo X, Cao B. 2023. “Superbugs” with hypervirulence and carbapenem resistance in Klebsiella pneumoniae: the rise of such emerging nosocomial pathogens in China. Sci Bull (Beijing) 68:2658–2670. doi:10.1016/j.scib.2023.09.040 37821268

[3] Yang X, Dong N, Chan EW-C, Zhang R, Chen S. 2021. Carbapenem resistance-encoding and virulence-encoding conjugative plasmids in Klebsiella pneumoniae. Trends Microbiol 29:65–83. doi:10.1016/j.tim.2020.04.012 32448764

[4] Papa-Ezdra R, Cordeiro NF, Ferreira F, García-Fulgueiras V, Araújo L, Mota MI, Outeda M, Seija V, Vignoli R, Bado I. 2024. First detection of high-level aminoglycoside-resistant Klebsiella pneumoniae and Enterobacter cloacae isolates due to 16S rRNA methyltransferases with and without blaNDM in Uruguay. Antibiotics (Basel) 13:1029. doi:10.3390/antibiotics13111029 39596724 PMC11590977

[5] Roberts T, Ling CL, Watthanaworawit W, Cheav C, Sengduangphachanh A, Silisouk J, Hopkins J, Phommasone K, Batty EM, Turner P, Ashley EA. 2024. AmpC beta-lactamases detected in southeast Asian Escherichia coli and Klebsiella pneumoniae. JAC Antimicrob Resist dlae195. doi:10.1093/jacamr/dlae195 PMC11604056 39610980

[6] Moradi F, Akbari M, Vakili-Ghartavol R, Ostovari M, Hadi N. 2024. Molecular characterization of superbugs K. pneumoniae harboring extended-spectrum β-lactamase (ESBL) and carbapenemase resistance genes among hospitalized patients in southwestern Iran, Western Asia. Heliyon 10:e36858. doi:10.1016/j.heliyon.2024.e36858 39263100 PMC11388783

[7] Lei TY, Liao BB, Yang LR, Wang Y, Chen XB. 2024. Hypervirulent and carbapenem-resistant Klebsiella pneumoniae: a global public health threat. Microbiol Res 288:127839. doi:10.1016/j.micres.2024.127839 39141971

[8] Lee CR, Lee JH, Park KS, Jeon JH, Kim YB, Cha CJ, Jeong BC, Lee SH. 2017. Antimicrobial resistance of hypervirulent Klebsiella pneumoniae: epidemiology, hypervirulence-associated determinants, and resistance mechanisms. Front Cell Infect Microbiol 7:483. doi:10.3389/fcimb.2017.00483 29209595 PMC5702448

[9] Kao CY, Kuo PY, Lin CC, Cheng YY, Wang MC, Chen YC, Lin WH. 2024. Molecular characterization of colistin-resistant Klebsiella pneumoniae isolates and their conjugative mcr-carrying plasmids. J Infect Public Health 17:102588. doi:10.1016/j.jiph.2024.102588 39566127

[10] Bakhtiyari N, Farajnia S, Ghasemali S, Farajnia S, Pormohammad A, Saeidvafa S. 2024. Strategies to overcome antimicrobial resistance in nosocomial infections, a review and update. Infect Disord Drug Targets 24:e260124226226. doi:10.2174/0118715265276529231214105423 38284691

[11] Summers WC. 2012. The strange history of phage therapy. Bacteriophage 2:130–133. doi:10.4161/bact.20757 23050223 PMC3442826

[12] Hatfull GF, Dedrick RM, Schooley RT. 2022. Phage therapy for antibiotic-resistant bacterial infections. Annu Rev Med 73:197–211. doi:10.1146/annurev-med-080219-122208 34428079

[13] Olawade DB, Fapohunda O, Egbon E, Ebiesuwa OA, Usman SO, Faronbi AO, Fidelis SC. 2024. Phage therapy: a targeted approach to overcoming antibiotic resistance. Microb Pathog 197:107088. doi:10.1016/j.micpath.2024.107088 39477033

[14] Cano EJ, Caflisch KM, Bollyky PL, Van Belleghem JD, Patel R, Fackler J, Brownstein MJ, Horne B, Biswas B, Henry M, Malagon F, Lewallen DG, Suh GA. 2021. Phage therapy for limb-threatening prosthetic knee Klebsiella pneumoniae infection: case report and in vitro characterization of anti-biofilm activity. Clin Infect Dis 73:e144–e151. doi:10.1093/cid/ciaa705 32699879 PMC8246933

[15] Corbellino M, Kieffer N, Kutateladze M, Balarjishvili N, Leshkasheli L, Askilashvili L, Tsertsvadze G, Rimoldi SG, Nizharadze D, Hoyle N, Nadareishvili L, Antinori S, Pagani C, Scorza DG, Romanò ALL, Ardizzone S, Danelli P, Gismondo MR, Galli M, Nordmann P, Poirel L. 2020. Eradication of a multidrug-resistant, carbapenemase-producing Klebsiella pneumoniae isolate following oral and intra-rectal therapy with a custom made, lytic bacteriophage preparation. Clin Infect Dis 70:1998–2001. doi:10.1093/cid/ciz782 31414123

[16] Labrie SJ, Samson JE, Moineau S. 2010. Bacteriophage resistance mechanisms. Nat Rev Microbiol 8:317–327. doi:10.1038/nrmicro2315 20348932

[17] Kuipers S, Ruth MM, Mientjes M, de Sévaux RGL, van Ingen J. 2019. A Dutch case report of successful treatment of chronic relapsing urinary tract infection with bacteriophages in a renal transplant patient. Antimicrob Agents Chemother 64:e01281-19. doi:10.1128/AAC.01281-19 31611357 PMC7187595

[18] Zhou F, Wang K, Ji S, Liao X, Zhang W, Teng T, Wang L, Li Q. 2025. The virulent bacteriophage Henu8 as an antimicrobial synergist against Escherichia coli. Microbiol Spectr 13:e0163324. doi:10.1128/spectrum.01633-24 40377308 PMC12210894

[19] Subedi D, Gordillo Altamirano F, Deehan R, Perera A, Patwa R, Kostoulias X, Korneev D, Blakeway L, Macesic N, Peleg AY, Barr JJ. 2025. Rational design of a hospital-specific phage cocktail to treat Enterobacter cloacae complex infections. Nat Microbiol 10:2702–2719. doi:10.1038/s41564-025-02130-4 40993246 PMC12578640

[20] Ellis EL, Delbrück M. 1939. The growth of bacteriophage. J Gen Physiol 22:365–384. doi:10.1085/jgp.22.3.365 19873108 PMC2141994

[21] Summer EJ. 2009. Preparation of a phage DNA fragment library for whole genome shotgun sequencing. Methods Mol Biol 502:27–46. doi:10.1007/978-1-60327-565-1_4 19082550

[22] Salih H, Karaynir A, Yalcin M, Oryasin E, Holyavkin C, Basbulbul G, Bozdogan B. 2022. Metagenomic analysis of wastewater phageome from a University hospital in Turkey. Arch Microbiol 204:353. doi:10.1007/s00203-022-02962-2 35637399

[23] Bolger AM, Lohse M, Usadel B. 2014. Trimmomatic: a flexible trimmer for Illumina sequence data. Bioinformatics 30:2114–2120. doi:10.1093/bioinformatics/btu170 24695404 PMC4103590

[24] Coil D, Jospin G, Darling AE. 2015. A5-miseq: an updated pipeline to assemble microbial genomes from Illumina MiSeq data. Bioinformatics 31:587–589. doi:10.1093/bioinformatics/btu661 25338718

[25] Bankevich A, Nurk S, Antipov D, Gurevich AA, Dvorkin M, Kulikov AS, Lesin VM, Nikolenko SI, Pham S, Prjibelski AD, Pyshkin AV, Sirotkin AV, Vyahhi N, Tesler G, Alekseyev MA, Pevzner PA. 2012. SPAdes: a new genome assembly algorithm and its applications to single-cell sequencing. J Comput Biol 19:455–477. doi:10.1089/cmb.2012.0021 22506599 PMC3342519

[26] Lowe TM, Chan PP. 2016. tRNAscan-SE on-line: integrating search and context for analysis of transfer RNA genes. Nucleic Acids Res 44:W54–W57. doi:10.1093/nar/gkw413 27174935 PMC4987944

[27] Wang RH, Yang S, Liu Z, Zhang Y, Wang X, Xu Z, Wang J, Li SC. 2024. PhageScope: a well-annotated bacteriophage database with automatic analyses and visualizations. Nucleic Acids Res 52:D756–D761. doi:10.1093/nar/gkad979 37904614 PMC10767790

[28] Millard A, Denise R, Lestido M, Thomas MT, Webster D, Turner D, Sicheritz-Pontén T. 2025. taxMyPhage: automated taxonomy of dsDNA phage genomes at the genus and species level. PHAGE 6:5–11. doi:10.1089/phage.2024.0050 40351403 PMC12060842

[29] Grant JR, Enns E, Marinier E, Mandal A, Herman EK, Chen C, Graham M, Van Domselaar G, Stothard P. 2023. Proksee: in-depth characterization and visualization of bacterial genomes. Nucleic Acids Res 51:W484–W492. doi:10.1093/nar/gkad326 37140037 PMC10320063

[30] Guo W, Yang Z, Wang K, Li W, Zhao Y, Yang Y, Chang W, Gong Z, Liu Z, Chen Y, Li Q. 2024. Discovery of unique bis-substituted aromatic amide derivatives as novel highly potent antibiotics for combating methicillin-resistant Staphylococcus aureus (MRSA). J Med Chem 67:2129–2151. doi:10.1021/acs.jmedchem.3c02064 38289145

[31] Li Q, Li J, Zhao Y, Guo S, Liu M, Shi X, Wang L, Liu Z, Teng T. 2025. Characterization and genomics of phage Henu2_3 against K1 Klebsiella pneumoniae and its efficacy in animal models. AMB Express 15:112. doi:10.1186/s13568-025-01919-0 40736875 PMC12311074

[32] Brisse S, Passet V, Haugaard AB, Babosan A, Kassis-Chikhani N, Struve C, Decré D. 2013. Wzi gene sequencing, a rapid method for determination of capsular type for Klebsiella strains. J Clin Microbiol 51:4073–4078. doi:10.1128/JCM.01924-13 24088853 PMC3838100

[33] Piperaki ET, Syrogiannopoulos GA, Tzouvelekis LS, Daikos GL. 2017. Klebsiella pneumoniae: virulence, biofilm and antimicrobial resistance. Pediatr Infect Dis J 36:1002–1005. doi:10.1097/INF.0000000000001675 28914748

[34] Kunisch F, Campobasso C, Wagemans J, Yildirim S, Chan BK, Schaudinn C, Lavigne R, Turner PE, Raschke MJ, Trampuz A, Gonzalez Moreno M. 2024. Targeting Pseudomonas aeruginosa biofilm with an evolutionary trained bacteriophage cocktail exploiting phage resistance trade-offs. Nat Commun 15:8572. doi:10.1038/s41467-024-52595-w 39362854 PMC11450229

[35] Mayorga-Ramos A, Carrera-Pacheco SE, Barba-Ostria C, Guamán LP. 2024. Bacteriophage-mediated approaches for biofilm control. Front Cell Infect Microbiol 14:1428637. doi:10.3389/fcimb.2024.1428637 39435185 PMC11491440

[5] Ménard G, Rouillon A, Cattoir V, Donnio P-Y. 2021. Galleria mellonella as a suitable model of bacterial infection: past, present and future. Front Cell Infect Microbiol 11:782733. doi:10.3389/fcimb.2021.782733 35004350 PMC8727906

[37] Zhang P, Zeng P, Lai CKC, Ip M, To KKW, Zuo Z, Xia J, Leung SSY. 2024. Synergism of colistin and globular endolysins against multidrug-resistant Gram-negative bacteria. Int J Biol Macromol 278:134670. doi:10.1016/j.ijbiomac.2024.134670 39151868

[38] Han P, Pu M, Li Y, Fan H, Tong Y. 2023. Characterization of bacteriophage BUCT631 lytic for K1 Klebsiella pneumoniae and its therapeutic efficacy in Galleria mellonella larvae. Virol Sin 38:801–812. doi:10.1016/j.virs.2023.07.002 37419417 PMC10590696

[39] Malathi K, Anbarasu A, Ramaiah S. 2019. Identification of potential inhibitors for Klebsiella pneumoniae carbapenemase-3: a molecular docking and dynamics study. J Biomol Struct Dyn 37:4601–4613. doi:10.1080/07391102.2018.1556737 30632921

[40] Abbas R, Chakkour M, Zein El Dine H, Obaseki EF, Obeid ST, Jezzini A, Ghssein G, Ezzeddine Z. 2024. General overview of Klebsiella pneumonia: epidemiology and the role of siderophores in its pathogenicity. Biology (Basel) 13:78. doi:10.3390/biology13020078 38392297 PMC10886558

[41] Ding L, Shen S, Chen J, Tian Z, Shi Q, Han R, Guo Y, Hu F. 2023. Klebsiella pneumoniae carbapenemase variants: the new threat to global public health. Clin Microbiol Rev 36:e0000823. doi:10.1128/cmr.00008-23 37937997 PMC10732083

[42] Casanova C, Lorente JA, Carrillo F, Pérez-Rodríguez E, Núñez N. 1989. Klebsiella pneumoniae liver abscess associated with septic endophthalmitis. Arch Intern Med 149:1467. 2658901

[43] Russo TA, Olson R, Fang C-T, Stoesser N, Miller M, MacDonald U, Hutson A, Barker JH, La Hoz RM, Johnson JR, Backer M, Bajwa R, Catanzaro AT, Crook D, de Almeda K, Fierer J, Greenberg DE, Klevay M, Patel P, Ratner A, Wang J-T, Zola J. 2018. Identification of biomarkers for differentiation of hypervirulent Klebsiella pneumoniae from Classical K. pneumoniae. J Clin Microbiol 56. doi:10.1128/JCM.00776-18 PMC6113484 29925642

[44] Yeh KM, Kurup A, Siu LK, Koh YL, Fung CP, Lin JC, Chen TL, Chang FY, Koh TH. 2007. Capsular serotype K1 or K2, rather than magA and rmpA, is a major virulence determinant for Klebsiella pneumoniae liver abscess in Singapore and Taiwan. J Clin Microbiol 45:466–471. doi:10.1128/JCM.01150-06 17151209 PMC1829066

[45] Fung C-P, Chang F-Y, Lee S-C, Hu B-S, Kuo BI-T, Liu C-Y, Ho M, Siu LK. 2002. A global emerging disease of Klebsiella pneumoniae liver abscess: is serotype K1 an important factor for complicated endophthalmitis? Gut 50:420–424. doi:10.1136/gut.50.3.420 11839725 PMC1773126

[46] Hala S, Malaikah M, Huang J, Bahitham W, Fallatah O, Zakri S, Antony CP, Alshehri M, Ghazzali RN, Ben-Rached F, Alsahafi A, Alsaedi A, AlAhmadi G, Kaaki M, Alazmi M, AlhajHussein B, Yaseen M, Zowawi HM, Alghoribi MF, Althaqafi AO, Al-Amri A, Moradigaravand D, Pain A. 2024. The emergence of highly resistant and hypervirulent Klebsiella pneumoniae CC14 clone in a tertiary hospital over 8 years. Genome Med 16:58. doi:10.1186/s13073-024-01332-5 38637822 PMC11025284

[47] Gálvez-Silva M, Arros P, Berríos-Pastén C, Villamil A, Rodas PI, Araya I, Iglesias R, Araya P, Hormazábal JC, Bohle C, Chen Y, Gan Y-H, Chávez FP, Lagos R, Marcoleta AE. 2024. Carbapenem-resistant hypervirulent ST23 Klebsiella pneumoniae with a highly transmissible dual-carbapenemase plasmid in Chile. Biol Res 57:7. doi:10.1186/s40659-024-00485-2 38475927 PMC10929235

[48] Wen Z, Chen Y, Liu T, Han J, Jiang Y, Zhang K. 2024. Predicting antibiotic tolerance in hvKP and cKP respiratory infections through biofilm formation analysis and its resistance implications. Infect Drug Resist 17:1529–1537. doi:10.2147/IDR.S449712 38650753 PMC11033731

[49] Taha MS, Elkolaly RM, Elhendawy M, Elatrozy H, Amer AF, Helal RAEF, Salem H, El Feky YG, Harkan A, Mashaal RG, Allam AA, Oraiby AE, Abdeen NSM, Bahey MG. 2024. Phenotypic and genotypic detection of hypervirulent Klebsiella pneumoniae isolated from hospital-acquired infections. Microorganisms 12:2469. doi:10.3390/microorganisms12122469 39770672 PMC11728040

[50] Colavecchio A, Cadieux B, Lo A, Goodridge LD. 2017. Bacteriophages contribute to the spread of antibiotic resistance genes among foodborne pathogens of the Enterobacteriaceae family – a review. Front Microbiol 8:1108. doi:10.3389/fmicb.2017.01108 28676794 PMC5476706

[51] Xi H, Fu B, Sheng Q, Luo M, Sun L. 2024. Isolation and characterization of a lytic bacteriophage RH-42-1 of Erwinia amylovora from orchard soil in China. Viruses 16:509. doi:10.3390/v16040509 38675852 PMC11054837

[52] Gulati P, Singh A, Patra S, Bhat S, Verma A. 2024. Restriction modification systems in archaea: a panoramic outlook. Heliyon 10:e27382. doi:10.1016/j.heliyon.2024.e27382 38644887 PMC11033074

[53] Dunstan RA, Bamert RS, Tan KS, Imbulgoda U, Barlow CK, Taiaroa G, Pickard DJ, Schittenhelm RB, Dougan G, Short FL, Lithgow T. 2023. Epitopes in the capsular polysaccharide and the porin OmpK36 receptors are required for bacteriophage infection of Klebsiella pneumoniae. Cell Rep 42:112551. doi:10.1016/j.celrep.2023.112551 37224021

[54] Squeglia F, Maciejewska B, Łątka A, Ruggiero A, Briers Y, Drulis-Kawa Z, Berisio R. 2020. Structural and functional studies of a Klebsiella phage capsule depolymerase tailspike: mechanistic insights into capsular degradation. Structure 28:613–624. doi:10.1016/j.str.2020.04.015 32386574

[55] Pires DP, Oliveira H, Melo LDR, Sillankorva S, Azeredo J. 2016. Bacteriophage-encoded depolymerases: their diversity and biotechnological applications. Appl Microbiol Biotechnol 100:2141–2151. doi:10.1007/s00253-015-7247-0 26767986

[56] Zhao R, Jiang S, Ren S, Yang L, Han W, Guo Z, Gu J. 2024. A novel phage putative depolymerase, Depo16, has specific activity against K1 capsular-type Klebsiella pneumoniae. Appl Environ Microbiol 90:e0119723. doi:10.1128/aem.01197-23 38551353 PMC11022553

[57] Wu Y, Wang R, Xu M, Liu Y, Zhu X, Qiu J, Liu Q, He P, Li Q. 2019. A novel polysaccharide depolymerase encoded by the phage SH-KP152226 confers specific activity against multidrug-resistant Klebsiella pneumoniae via biofilm degradation. Front Microbiol 10:2768. doi:10.3389/fmicb.2019.02768 31849905 PMC6901502

[58] Li M, Wang H, Chen L, Guo G, Li P, Ma J, Chen R, Du H, Liu Y, Zhang W. 2022. Identification of a phage-derived depolymerase specific for KL47 capsule of Klebsiella pneumoniae and its therapeutic potential in mice. Virol Sin 37:538–546. doi:10.1016/j.virs.2022.04.005 35513275 PMC9437526

[59] Li Y, Pu M, Han P, Li M, An X, Song L, Fan H, Chen Z, Tong Y. 2023. Efficacy in Galleria mellonella larvae and application potential assessment of a new bacteriophage BUCT700 extensively lyse Stenotrophomonas maltophilia. Microbiol Spectr 11:e0403022. doi:10.1128/spectrum.04030-22 36700630 PMC9927281

[60] Senhaji-Kacha A, Bernabéu-Gimeno M, Domingo-Calap P, Aguilera-Correa JJ, Seoane-Blanco M, Otaegi-Ugartemendia S, van Raaij MJ, Esteban J, García-Quintanilla M. 2024. Isolation and characterization of two novel bacteriophages against carbapenem-resistant Klebsiella pneumoniae. Front Cell Infect Microbiol 14:1421724. doi:10.3389/fcimb.2024.1421724 39268483 PMC11390652

[61] Cai R, Wu M, Zhang H, Zhang Y, Cheng M, Guo Z, Ji Y, Xi H, Wang X, Xue Y, Sun C, Feng X, Lei L, Tong Y, Liu X, Han W, Gu J. 2018. A smooth-type, phage-resistant Klebsiella pneumoniae mutant strain reveals that OmpC is indispensable for infection by phage GH-K3. Appl Environ Microbiol 84. doi:10.1128/AEM.01585-18 PMC6193389 30171001

[62] Karthika C, Malligarjunan N, Hari Prasath N, Karutha Pandian S, Gowrishankar S. 2025. Phage (cocktail)-antibiotic synergism: a new frontier in addressing Klebsiella pneumoniae resistance. Front Microbiol 16:1588472. doi:10.3389/fmicb.2025.1588472 40400679 PMC12092377

[63] Martinez-Soto CE, McClelland M, Kropinski AM, Lin JT, Khursigara CM, Anany H. 2024. Multireceptor phage cocktail against Salmonella enterica to circumvent phage resistance. Microlife 5:uqae003. doi:10.1093/femsml/uqae003 38545601 PMC10972627

[64] Plumet L, Costechareyre D, Lavigne JP, Kissa K, Molle V. 2024. Zebrafish as an effective model for evaluating phage therapy in bacterial infections: a promising strategy against human pathogens. Antimicrob Agents Chemother 68:e0082924. doi:10.1128/aac.00829-24 39248472 PMC11460995

[65] Fang C, Dai X, Xiang L, Qiu Y, Yin M, Fu Y, Li Y, Zhang L. 2023. Isolation and characterization of three novel lytic phages against K54 serotype carbapenem-resistant hypervirulent Klebsiella pneumoniae. Front Cell Infect Microbiol 13:1265011. doi:10.3389/fcimb.2023.1265011 38149011 PMC10749971

[66] Manohar P, Nachimuthu R, Lopes BS. 2018. The therapeutic potential of bacteriophages targeting Gram-negative bacteria using Galleria mellonella infection model. BMC Microbiol 18:97. doi:10.1186/s12866-018-1234-4 30170558 PMC6119258

