# Supplementary material for: Characterization of the bacteriophage vB_KpnP_Henu1_3 lytic for K1 Klebsiella pneumoniae and its therapeutic efficacy in Galleria mellonella larvae and mice
Source: Microbiol Spectr. 2026 Jan 6;14(2):e00931-25. doi: 10.1128/spectrum.00931-25 (PMC12889090; doi:10.1128/spectrum.00931-25)
Supplement: Tables S1 — The isolation information of clinical isolates used in this study. [file spectrum.00931-25-s0001.doc]

**TABLE S1** The isolation information of clinical isolates used in this study

| Species | Strains name | Capsular type  (K-loci) | Sources of isolation | Antibiotic resistance profiles | Isolation date |
| --- | --- | --- | --- | --- | --- |
| *K. pneumoniae* | Kp1049 | K1 | Sewage | Ampicillin | 2023/12/01 |
|  | Kp0311 | K1 | Sputa | Ampicillin | 2024/09/05 |
|  | Kp0822 | K1 | Sputa | Ampicillin | 2024/11/09 |
|  | Kp407 | K1 | Sputa | Ampicillin | 2024/09/12 |
|  | Kp0918 | K1 | Sputa | Ampicillin | 2024/12/10 |
|  | Kp408 | K2 | Sputa | Ampicillin | 2024/08/25 |
|  | Kp1203 | K2 | Sputa | Ampicillin | 2024/09/13 |
|  | Kp1616 | K2 | Sputa | Ampicillin | 2024/10/17 |
|  | Kp0524 | K2 | Sputa | Ampicillin, cefazolin | 2024/08/06 |
|  | Kp1904-2431 | K19 | Sputa | Ampicillin, Ciprofloxacin, levofloxacin, ceftriaxone, gentamicin, chloramphenicol | 2022/05/23 |
|  | Kp302 | K19 | Sputa | Ampicillin | 2024/10/12 |
|  | Kp309 | K63 | Sputa | Ampicillin | 2024/09/12 |
|  | Kp403 | K62 | Sputa | Ampicillin | 2024/09/20 |
|  | Kp2640 | K28 | Sputa | Ampicillin, chloramphenicol | 2024/10/27 |
|  | Kp126N | K28 | Sputa | Ampicillin, cefazolin | 2024/09/25 |
|  | Kp306N | K14,K64 | Sputa | Ampicillin | 2024/10/12 |
|  | Kp347N | K14,K64 | Sputa | Ampicillin | 2024/10/13 |
|  | Kp2001-0185 | K14,K64 | Sputa | Ampicillin, Ciprofloxacin, levofloxacin, ceftriaxone, gentamicin | 2022/08/09 |
|  | Kp2001-0219 | K14,K64 | Sputa | Ampicillin, Ciprofloxacin, levofloxacin, ceftriaxone, gentamicin | 2023/05/04 |
|  | Kp2011-3676 | K14,K64 | Sputa | Ampicillin, Ciprofloxacin, levofloxacin, ceftriaxone, gentamicin, chloramphenicol | 2023/06/23 |
|  | Kp1901-0124 | K14,K64 | Sputa | Ampicillin, Ciprofloxacin, levofloxacin, ceftriaxone, gentamicin | 2023/03/21 |
|  | Kp120804 | K62 | Sputa | Ampicillin, chloramphenicol | 2024/11/23 |
|  | Kp120819 | K63 | Sputa | Ampicillin | 2024/11/22 |
|  | Kp57N | K63 | Sputa | Ampicillin | 2025/02/23 |
|  | Kp0953 | K19 | Sputa | Ampicillin, Ciprofloxacin, levofloxacin, selectrin | 2024/11/10 |
|  | Kp0706 | K14 | Serum | Ampicillin | 2024/12/10 |
|  | Kp2828 | K16 | Sputa | Ampicillin, Ampicillin/Sulbactam | 2024/11/29 |
